# Early cortical GABAergic interneurons determine the projection patterns of L4 excitatory neurons

**DOI:** 10.1126/sciadv.adj9911

**Published:** 2024-05-10

**Authors:** Lorena Bragg-Gonzalo, Alfonso Aguilera, Candela González-Arias, Noelia S. De León Reyes, Alonso Sánchez-Cruz, Paula Carballeira, Félix Leroy, Gertrudis Perea, Marta Nieto

**Affiliations:** ^1^ Department of Molecular and Cellular Biology, Centro Nacional de Biotecnología, Consejo Superior de Investigaciones Científicas (CNB-CSIC), Madrid 28049, Spain.; ^2^ Functional and Systems Neurobiology Department, Instituto Cajal, Consejo Superior de Investigaciones Científicas, Madrid 28002, Spain.; ^3^ Instituto de Neurociencias (CSIC-UMH), Av. Ramón y Cajal s/n, San Juan de Alicante, Alicante, Spain.

## Abstract

During cerebral cortex development, excitatory pyramidal neurons (PNs) establish specific projection patterns while receiving inputs from GABAergic inhibitory interneurons (INs). Whether these inhibitory inputs can shape PNs’ projection patterns is, however, unknown. While layer 4 (L4) PNs of the primary somatosensory (S1) cortex are all born as long-range callosal projection neurons (CPNs), most of them acquire local connectivity upon activity-dependent elimination of their interhemispheric axons during postnatal development. Here, we demonstrate that precise developmental regulation of inhibition is key for the retraction of S1L4 PNs’ callosal projections. Ablation of somatostatin INs leads to premature inhibition from parvalbumin INs onto S1L4 PNs and prevents them from acquiring their barrel-restricted local connectivity pattern. As a result, adult S1L4 PNs retain interhemispheric projections responding to tactile stimuli, and the mice lose whisker-based texture discrimination. Overall, we show that temporally ordered IN activity during development is key to shaping local ipsilateral S1L4 PNs’ projection pattern, which is required for fine somatosensory processing.

## Teaser

Timely maturation of early inhibition ensures the elimination of developmental S1L4 callosal axons and fine-tuned somatosensation.

## INTRODUCTION

Excitatory pyramidal neurons (PNs) of the cerebral cortex constitute the building blocks of structural connectivity, while the less abundant inhibitory γ-aminobutyric acid (GABA)-ergic interneurons (INs) are crucial regulators of activity (*1*, *2*). INs comprise diverse types that selectively connect with the different PNs subclasses, mediating computation in the adult (*3*). Such specific IN-PN connectivity arises during postnatal development in precise temporal order. In parallel, PNs gradually differentiate and acquire subtype-specific projection patterns (*3*–*7*). However, whether the developmental emergence of IN inhibition influences PNs’ axonal wiring and, thus, the final structure of the adult circuit is still unknown.

**Fig. 1.**
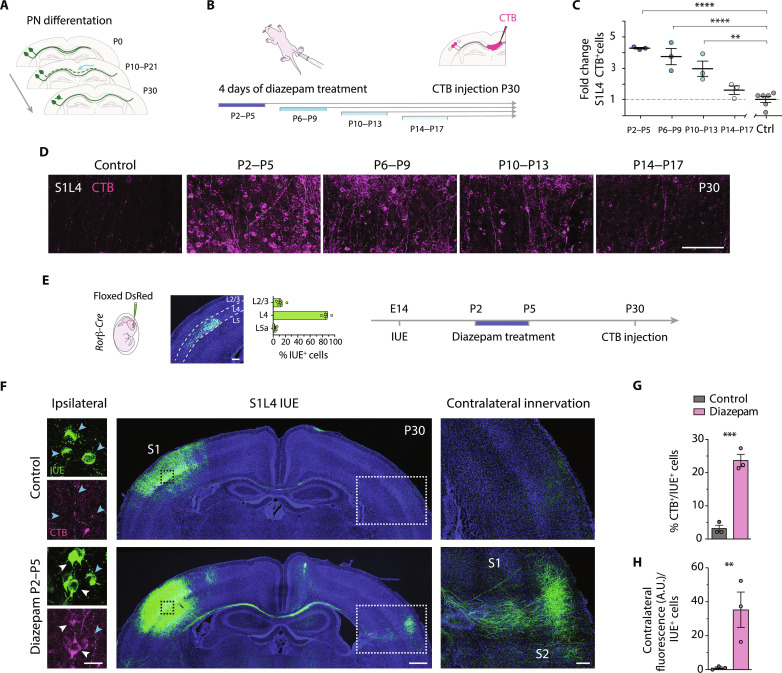
Diazepam induces S1L4 PN rewiring. (**A**) Developmental PNs’ callosal refinement. (**B**) Experimental design. Four groups of mice were daily treated with diazepam for four consecutive days in different temporal windows ranging from P2 to P17. After, CTB was injected directly into the CC at P30 to evaluate the adult callosal circuit. (**C**) Increases of P30 S1L4 CPNs after diazepam treatment (*n* ≥ 3 mice per condition). (**D**) Confocal images of P30 S1L4 CPNs (CTB^+^). (**E**) In utero electroporation (IUE) of *Ror*β-*Cre* embryos at E14 (left), layer distribution of IUE^+^ cells (middle) (DsRed fluorescence, green) (*n* = 600 cells), and procedure timeline (right). (**F**) Confocal magnifications and tilescans of IUE^+^ (green), CTB^+^ (magenta) cells, and callosal axons at P30. (**G**) Percentage of CPNs in electroporated neurons (*n* = 3 mice per condition). (**H**) Index of contralateral innervation (fluorescence signal in the contralateral cortex relative to ipsilateral IUE^+^ cells) (*n* = 3 mice per condition). Dots represent mice-individual values. Data are means ± SEM. **P < 0.01, ***P < 0.001, and ****P < 0.0001. Unpaired *t* test in (G) and (H) and one-way analysis of variance (ANOVA) followed by Dunnett’s multiple comparisons test in (C). Scale bars, 500 μm (F, middle), 200 μm (E), 100 μm (D and F, right), and 25 μm (F, left). A.U., arbitrary units.

Recent data demonstrate that all PNs in layer 2/3 (L2/3) and L4 initially project interhemispherically through the corpus callosum (CC) during differentiation (*8*). They all display a common early molecular identity that defines them as callosal projection neurons (CPNs) (*6*). Only during postnatal differentiation do these PNs eliminate or stabilize their developmental interhemispheric axons to mature as local or CPNs (Fig. 1A) (*6*, *8*). This selection is activity-dependent, area- and layer-specific, and instructed by the thalamus (*8*–*10*). Because cortical INs are early recipients of thalamic inputs (*11*–*14*), we investigated whether they influence PN callosal versus local wiring.

INs can be classified into over two dozen subclasses based on morphology, electrical responses, and gene expression (*3*, *15*, *16*). In the cerebral cortex, somatostatin (SST)– and parvalbumin (PV)–expressing INs (SST-INs and PV-INs, respectively) are the most abundant GABAergic subtypes (*2*). SST-INs are the first to operate in S1, establishing transient circuitries with PV-INs and S1L4 PNs (*11*–*13*). SST-IN temporary connections have been proven necessary for the timely maturation of thalamocortical (TC) inputs onto both PV-INs and S1L4 PN types (*11*, *12*). Once TC inputs are established onto their adult targets, PV-IN axons innervate L4 PNs to mediate feedforward inhibition (*14*, *17*). These studies crucially highlighted the IN-dependent mode of thalamic assembly onto the cortex. Herein, we show that SST-INs also orchestrate the wiring of S1 intracortical networks.

We previously demonstrated that TC inputs instruct the refinement of developmental callosal projections and define adult interhemispheric connectivity (*8*), but the mechanisms behind the process remained unclear. To analyze CPN selection further, our first approach was to manipulate activity broadly and systemically to identify conditions and temporal windows leading to changes in CPNs and then proceed to an in-depth investigation of the processes of CPN conversion. To deregulate activity systemically, we opted to treat mice during different postnatal windows with diazepam, a positive allosteric modulator of the γ-aminobutyric acid type A (GABA_A_) receptor (GABA_A_R), and then analyze the effect on adult CPNs. We found that treatments over the first and second postnatal weeks increased the number of adult S1L4 CPNs. This demonstrated that early activity-dependent wiring determines CPN differentiation. The increase in S1L4 CPNs in diazepam-treated animals was due to an increased rate of axonal stabilization during the refinement that typically eliminates S1L4 PN transient callosal projections. This change was not during the time of treatment but days after the treatment ended, indicating that early wiring prospectively modifies refinement. Because presynaptic connectivity is known to influence the stabilization of callosal axons and, therefore, would be a likely cause of rewiring, we characterized, immediately after ending the treatment, the changes diazepam created on S1L4 PN input connectivity. We found that diazepam had impaired the establishment of nascent excitatory synapses onto S1L4 PNs and had induced a reduction in thalamic vesicular glutamate transporter 2-positive (Vglut2^+^) contacts. This indicated that early reductions in excitatory inputs later facilitate S1L4 PN integration with contralateral networks. To investigate this hypothesis further, we searched for physiological regulators of S1L4 PN inputs and tested whether they could developmentally determine the wiring of the PNs. Because cortical SST-INs are active early and mediate the functional establishment of thalamic axons onto S1L4 PNs (*11*, *12*), we tested them as candidates to play this role. Using genetic ablations, we demonstrated that, in the absence of local SST-INs, ~25% of S1L4 PNs do not retract their developmental callosal projections and mature as adult S1L4 CPNs. We further showed that this lack of pruning was due to PV-IN premature inhibition of S1L4 PNs over the first postnatal week. This inhibition functionally decreased S1L4 PN responses to excitatory stimuli. It mimicked the early disengaging of S1L4 PNs from the thalamus observed with diazepam. Together, the experiments supported that early connectivity is responsible for a later selection of local connectivity. We also demonstrated that noncanonical interhemispheric axons from S1L4 CPNs induced upon SST-INs ablations are active during object exploration and that these mice lose whisker-mediated somatosensation. Overall, our results demonstrate that maturation of the barrel cortex and fine sensory perception requires an SST-IN-mediated elimination of the potential contralateral connectivity of L4 PNs. They demonstrate an essential role of INs in shaping the adult projection patterns of PNs and provide an extended and more comprehensive view of cortical wiring and the etiology of psychiatric and neurodevelopmental disorders (NDDs).

## RESULTS

### Diazepam treatment increases S1L4 CPNs

We sought to test whether altering GABA signaling can influence CPN development. To do so, we performed intraperitoneal injections of the GABA_A_R allosteric modulator diazepam. Animals were injected daily during different postnatal day (P) windows: P2 to P5, P6 to P9, P10 to P13, and P14 to P17. The effect of GABA on mature CPN numbers was then evaluated by injecting the retrograde tracer cholera toxin subunit B (CTB) into the CC at P30 (Fig. 1B and fig. S1A). This tracing strategy labels the soma of all PNs with a callosal axon at the time of injection. It shows much higher labeling efficiency than the classical injections in the gray matter because it reports all types of callosal projections, including the developmental projections that do not enter and branch into the cortical plate (*8*). In the canonical adult S1 circuit, most S1L4 neurons are local. Accordingly, injecting with CTB untreated animals in the CC at P30 led to few cells in S1L4 being labeled with the tracer (Fig. 1D). In contrast, several diazepam treatments increased the number of CTB^+^ cells in S1L4 (Fig. 1, C and D). The earliest treatment window (P2 to P5) was the most efficient, showing a fourfold increase in S1L4 CPNs (24 ± 0.5% CTB^+^ cells in diazepam-treated against 6 ± 2.65% in untreated controls) (Fig. 1C and fig. S1, B to D). Notably, the number of induced S1L4 CPNs progressively diminished when diazepam treatment began at later time points until having no effect when starting at P14 (Fig. 1, C and D). Therefore, P14 marks the closure of S1L4 CPN plasticity.

As for effects in other layers, the P2 to P5 treatment led to a slight decrease in S1L2/3 CPNs, while CPNs in S1L5 and S1L6 always remained unchanged (fig. S1D). We also analyzed the effects in the secondary somatosensory (S2) and primary visual cortex (V1) of the earliest and most effective treatment (P2 to P5). Diazepam did not alter CPN proportions in these areas (fig. S1, E and F). We then tested whether a prolonged diazepam treatment from P4 to P12 might show more potent effects, especially in the later-maturing S2 and V1 areas (fig. S1G). This extended treatment had similar effects to the P2 to P5 treatment (fig. S1H), i.e., incrementing S1L4 CPNs without affecting other S1 layers. It also failed to change the proportion of CPNs in S2 (fig. S1I) or V1 (fig. S1J). Hence, systemic activation of GABA_A_R signaling during the first 2 weeks of postnatal development has layer- and area-specific effects. Such differential impact is likely due to differences in the developmental time of maturation and spatial and cellular organization of circuits in S1, S2, and V1 areas (*18*, *19*).

To understand the mechanisms behind the selection between callosal and local wiring, we first examined the effects of diazepam on the axonal behavior of S1L4 PNs. To genetically label L4 PNs, we introduced a plasmid encoding a floxed fluorescent reporter into the cortex of *retinoic acid-related orphan receptor beta* (*Ror*β-*Cre*) mice (*8*, *20*) by in utero electroporation (IUE) at embryonic day 14 (E14) (Fig. 1E and fig. S2, A to E). Then, we treated the electroporated pups daily with diazepam from P2 to P5 and analyzed the mice at P30 to visualize the rewired electroporated PNs. IUE^+^ S1L4 PNs of diazepam-treated mice showed the expected increases in CTB^+^ labeling (S1L4 CPNs) compared to untreated animals (23 ± 3.2% against 3 ± 1.7%) (Fig. 1, F and G). The increment in CTB labeling was accompanied by a dense contralateral column of ramified callosal axons at the S1/S2 border, while S1L4 PNs of untreated animals display the typical ipsilateral restricted projections (Fig. 1, F and H, and fig. S2A). Quantifications of the columnar fluorescence signal and ipsilateral IUE^+^ cells demonstrated that the column was the consequence of an increased number of CPNs and not due to differential neuronal survival or electroporation efficiency (Fig. 1H and fig. S2, B to E). The results thus indicate that diazepam-induced S1L4 CPNs elaborate mature axonal branches in the contralateral hemisphere.

**Fig. 2.**
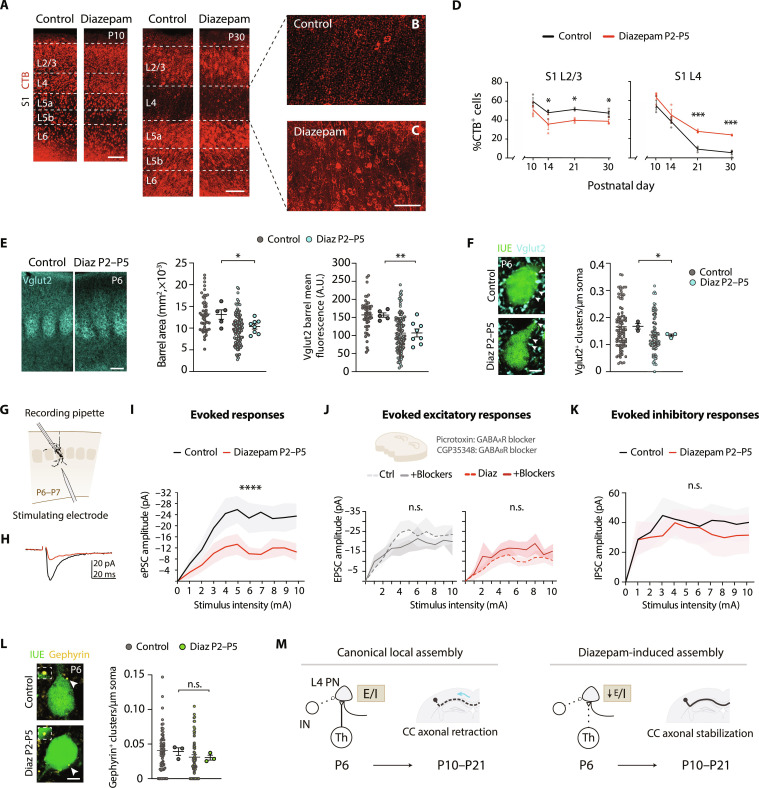
Characterization of diazepam-induced rewiring. (**A** to **C**) Confocal images of P10 (left) and P30 (right) S1 CPNs after P2 to P5 diazepam treatment. (**D**) Quantification of S1L2/3 and S1L4 CPNs at P10, P14, P21, and P30 after P2 to P5 diazepam treatment (*n* ≥ 3 mice per condition). (**E**) Vglut2 staining (left) and measurements of barrel size (middle) and barrel mean fluorescence (right) at P6. Small dots represent individual barrel values, whereas big dots show average value per mouse (*n* ≥ 49 barrels and *n* ≥ 5 mice per condition). (**F**) Vglut2^+^ boutons in P6 S1L4 IUE^+^ cells. Small dots represent individual cell values, whereas big dots show average value per mouse (*n* ≥ 73 cells, *n* = 3 mice per condition). (**G** to **I**) Synaptic response of S1L4 PNs’ to deep-layer stimulation (*n* ≥ 7 cells and *n* = 3 mice per condition). (**J**) Effect of GABA receptor blockage as in (G) to (I) (*n* ≥ 8 cells and *n* = 3 mice per condition). (**K**) Inhibitory synaptic response of S1L4 PNs’ to deep-layer stimulation as in (G) to (I) in the presence of NMDA and AMPA/kainate blockers (*n* ≥ 9 cells and *n* = 3 mice per condition). (**L**) Perisomatic gephyrin clusters on P6 S1L4 IUE^+^ cells. Small dots represent individual cell values, whereas big dots show average value per mouse (*n* = 59 cells and *n* = 3 mice per condition). (**M**) Proposed model of S1L4 PNs rewiring. The early failure in engaging ipsilateral innervation facilitates a later stabilization of S1L4 developmental callosal projections when they reach the contralateral hemisphere. Data are means ± SEM. Solid lines represent means and shaded area ± SEM. *P < 0.05, **P < 0.01, ***P < 0.001, and ****P < 0.0001; n.s., nonsignificant. Unpaired *t* test (D to F and L) and two-way ANOVA followed by Šídák’s multiple comparisons test in (I to K). Scale bars, 100 μm (A and E), 50 μm (B and C), and 2.5 μm (F and L).

Next, we studied changes across different postnatal stages resulting from P2 to P5 diazepam treatment. In a canonical scenario, S1L4 transient interhemispheric axons grow during P1 to P7 and undergo a steady elimination from P10 to P21 (*8*, *21*). In electroporated animals, analyzed at P6, IUE^+^ callosal axons from L4 showed similar trajectories, lengths, and midline occupancy in control and treated animals (fig. S2, F to J). Thus, diazepam neither reroutes developmental L4 callosal axons nor accelerates their invasion of contralateral territories. In addition, quantifications of IUE^+^ cells in this experiment further confirmed that diazepam does not alter P1-P5 developmental death (fig. S2G). After this analysis, we examined the dynamics of axonal elimination in control and diazepam-treated mice by injecting, in separated animals, CTB in the CC at representative stages ranging from P10 to P30. The experiment revealed that P2 to P5 diazepam treatment diminishes the high rates of developmental axonal refinement that distinguish S1L4 PNs canonical wiring (Fig. 2, A to D) (*8*). Thus, increased axonal stabilization explains the resulting increments in P30 S1L4 CPNs. Notably, although the treatment is taking place in the first postnatal week, the change in refinement is apparent only after P14 (Fig. 2D). Furthermore, quantification of S1L2/3 CTB^+^ cells in the same experiment confirmed the opposite dynamic in S1L2/3 PNs, for which augmented rates of CPN elimination after P14 led to the decrease in S1L2/3 CPNs we observed in the adult (Fig. 2, A to D). S2L2/3 CPNs showed accelerated refinement, in this case ending with similar numbers of CPNs between groups at P30 (fig. S1E). Thus, diazepam does not alter the initial growth of callosal axons but modifies the axonal refinement that occurs several days after treatment completion in a layer- and area-specific manner.

Last, we evaluated possible changes of neuronal identity linked to CPN conversion. We labeled cortical sections for representative layer-specific transcription factors (TFs): chicken ovalbumin upstream promoter TF-interacting protein 2 (Ctip2) for subcortical projection neurons (*22*), cut-like homeobox 1 (Cux1) (*23*) for L2/3 and L4, and Rorβ as a marker of L3, L4, and L5A (*24*, *25*). We found no changes in the expression of any of the three markers in any cortical layer (fig. S3). Although other changes cannot be discarded, these results suggest that S1L4 interhemispheric wiring is not associated with a loss of principal L4 identity.

### P2 to P5 diazepam treatment impairs the development of excitatory inputs onto S1L4 neurons

The data showed that systemic deregulation of early GABA signaling prospectively changes callosal versus local wiring and affects axonal stabilization rates after the treatment. Interhemispheric wiring depends on presynaptic integration (*26*, *27*), and diazepam has been shown to promote changes in synaptic connectivity (*28*). Thus, we reasoned that the systemic changes induced by diazepam could collectively result in the alteration of S1L4 PN early ipsilateral connectivity, which would then explain CPN rewiring. Because our systemic treatment affects many different neuronal types and brain territories and could influence S1L4 PNs also indirectly, we decided not to focus on identifying a primary mechanism of action but on the final status of S1L4 PN inputs immediately after the treatment ends at P6.

First, we analyzed TC axons as these are the principal excitatory inputs of S1L4 PNs and migrate onto L4 precisely during P2 to P5 (*29*). TC axons form barrel-like structures that allow the topographic segregation of whisker information and can be visualized using Vglut2 immunostaining (*29*). At P6, in diazepam-treated animals, examination of the area and average intensity of immunofluorescence signal revealed smaller and less intense Vglut2^+^ barrels than in controls (Fig. 2E and fig. S4A). A more detailed analysis estimating the density of Vglut2^+^ boutons onto S1L4 IUE^+^ neurons confirmed reduced numbers of thalamic contacts per individual S1L4 PN (Fig. 2F), while Vglut2 clusters in L5A and L5B were unaffected (fig. S4, B and C). Analysis of vesicular glutamate transporter 1 (Vglut1), a marker of intracortical excitatory synapses, showed a similar, nonsignificant trend (fig. S4D). These results indicate that diazepam had impaired the establishment of TC inputs onto S1L4 PNs and suggest a similar, although less marked, effect on intracortical excitatory inputs impinging on S1L4 PNs.

After detecting structural alterations of TC inputs onto S1L4 PNs, we focused on the changes in functional connectivity resulting after diazepam treatment. For this, we performed whole-cell patch-clamp recordings of S1L4 PNs in acute brain slices. Taking into account the 22-hour half-life of diazepam (*30*), we waited for a minimum of 24 hours before performing the recordings to ensure no interferences of the drug. We observed no differences in intrinsic excitability or spontaneous synaptic activity of P6-P7 S1L4 PNs between diazepam-treated and control mice (fig. S4, E to H). Then, to further assess S1L4 synaptic integration within the developing local circuit, we measured evoked postsynaptic currents (ePSCs) upon electrical stimulation of S1L5/L6 (Fig. 2G). We placed the stimulating electrode at this location because, at P6-P7, most S1L4 PN inputs emerge from deep layers and the thalamus (*31*–*33*). Compared to controls, diazepam-treated S1L4 PNs exhibited significantly smaller ePSC amplitudes in response to a wide range of electrical stimulation intensities (Fig. 2, H and I, and fig. S4, I, J, and L).

We then evaluated the synaptic inhibitory tone contributing to ePSCs using bath applications of pharmacological GABA_A_ and GABA_B_ receptor (GABAR) blockers. Blockade of GABAR did not change evoked synaptic responses in control or diazepam-treated mice (Fig. 2J and fig. S4, K and M). This lack of effect fits with the reported relatively low number of inhibitory synapses in the cortex during the first postnatal week (*3*–*5*, *12*). To analyze the inhibitory component more precisely, we performed another set of recordings evaluating spontaneous and evoked inhibitory postsynaptic currents (IPSCs) at P6-P7. We again located the stimulation electrode in the deep layers as in our previous analyses. These experiments confirmed undistinguishable synaptic inhibition in P6-P7 control and diazepam-treated animals (Fig. 2K and fig. S5A). Hence, these analyses demonstrated that P2 to P5 diazepam treatment impairs the development of excitatory inputs onto S1L4 PNs with no detectable effect on the inhibitory drive.

In addition, to estimate synaptic strength, we analyzed the synaptic responses at the lowest step (1 mA) of our stimulation protocol (*34*). This analysis showed a tendency toward decreased synaptic efficacy (average of all responses including failures), synaptic potency (amplitude of responses), and probability of response (ratio of responses from the total of stimuli applied) in ePSCs from diazepam-treated animals (fig. S5, B to C). These results support possible reductions in excitatory synaptic strength concomitant with a decrease in the number of excitatory inputs. Similarly, regarding the strength of inhibitory synapses, our analysis at 1 mA detected a tendency to decrease synaptic potency and increased the probability of response in IPSCs in diazepam-treated animals (fig. S5D). This indicated that there could be effects of diazepam on the remodeling of IN inputs not detected in our global assessment with GABAR blockers. To further evaluate possible effects on IN networks, using *Sst-Cre* and *Pv-Cre* mice crossed with a floxed *td-Tomato* (*Tom*) reporter line, we quantified the number and layer distribution of *Sst*-Tom^+^ cells (fig. S6, A and B) and *Pv*-Tom^+^ cells (fig. S6C). We found no differences between control and diazepam-treated animals, discarding any effects of diazepam on the survival or migration of medial ganglionic eminence–derived INs (*35*). In addition, specific analysis of gephyrin, a structural postsynaptic marker of inhibitory synapses (*36*), at the perisoma of P6 S1L4 IUE^+^ PNs, showed indistinguishable low densities in control and diazepam-treated animals (Fig. 2L). This indicated that basket-IN inputs, which mature only later than P6 (*3*), are similarly underdeveloped in both conditions. Thus, we did not detect any changes in inhibition that could account for the reduced ePSCs in diazepam conditions. In conclusion, our analyses indicated that diazepam impairs the establishment of incoming excitatory inputs onto developing S1L4 PNs. This suggests that efficient synaptic integration of S1L4 PNs into intracortical and thalamic excitatory networks during an early critical window (P2 to P10) promotes the shedding of interhemispheric axons at later postnatal stages (Fig. 2M).

### Ipsilateral cortical SST-INs determine the canonical local wiring of S1L4 PNs

**Fig. 3.**
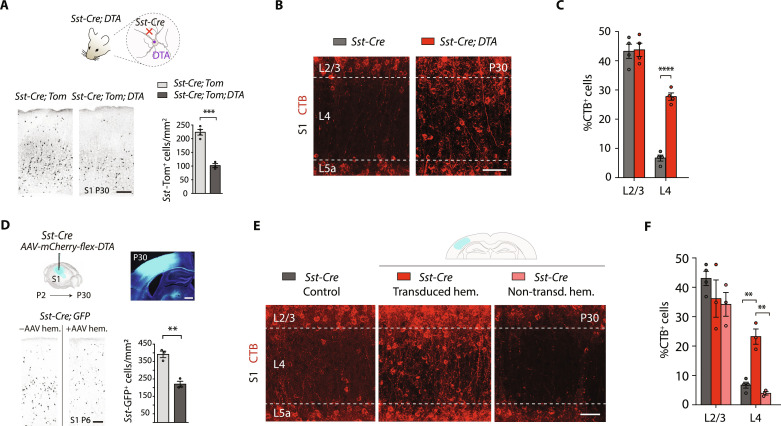
Somatostatin-IN ablation induces the callosal wiring of S1 L4 PNs. (**A**) Diagram (top) of the loss of SST-INs in *Sst*-*Cre*;*DTA*;*Tom* mice. Confocal images (bottom, left) and quantifications (bottom, right) at P30 of S1 SST-INs in *Sst*-*Cre*;*DTA*;*Tom* compared to control *Sst-Cre;Tom* mice (*n* ≥ 3 mice per condition). (**B**) Confocal images of CTB^+^ S1L4 CPNs. (**C**) Quantification of CPNs in S1 upper layers, showing increased numbers in S1L4 in mutant *Sst*-*Cre*;*DTA animals* (*n* = 4 mice per condition). (**D**) Loss of SST-INs in *Sst-Cre;GFP* mice after *AAV*-*mCherry-flex-DTA* injections at P2 in S1. Quantification at P6 in the S1 cortex (bottom, right) demonstrates a half-reduction in the density of SST-INs in transduced versus non-transduced animals (*n* = 3 mice). (**E**) Images of S1L4 CPNs in control and adeno-associated viral vector (AAV)–injected animals. (**F**) Quantification of CPNs in S1 upper-layers from (E). Only in the transduced S1 cortex, a significant higher number of S1L4 CPNs was observed. Dots represent mice-individual values. Data are means ± SEM. *n* ≥ 3 mice per condition. **P < 0.01, ***P < 0.001, and ****P < 0.0001. Unpaired *t* test in (A) and (D) and two-way ANOVA followed by Šídák’s multiple comparisons test in (C) and (F). Scale bars, 500 μm [(D), top)], 100 μm [(A) and (D), bottom], and 50 μm [(B) and (E)].

Given the critical contribution of excitatory inputs in shaping S1L4 PNs’ projection pattern, we next sought to investigate physiological regulators of early S1L4 PNs’ connectivity that could influence their adult wiring. GABAergic INs are principal regulators of cortical activity (*1*). They establish subclass-selective connections both with excitatory PN and IN subtypes during postnatal development (*3*). Among INs, SST-INs are the first to arrive, mature, and function in S1 (*11*–*13*). They are early recipients of thalamic connectivity and transiently innervate both PV-INs and S1L4 PNs, controlling the timely maturation of their TC inputs (*11*, *12*). To investigate a possible role of SST-INs in discarding potential CPN wiring, we genetically ablated SST-INs by crossing the *Sst*-*Cre* mouse line with a floxed *diphtheria toxin subunit A* (*DTA*) line (Fig. 3A). In these mice, SST-INs were specifically affected and reduced by half in S1 and S2 (Fig. 3A and fig. S7, A to H). The ablation induced a significant increase in the number of mature S1L4 CPNs (28 ± 2.5% in *Sst-Cre;DTA* mice compared to 7 ± 2.06% in *Sst-Cre*) (Fig. 3, B and C), while S1 CPNs in other layers and S2 CPNs remained unchanged (fig. S5, E and H). Moreover, crossing *Pv-Cre* mice with floxed *DTA* transgenic mice (fig. S7, I to K) did not alter the percentage of CPNs in S1 (fig. S7, L and M). As the genetic elimination of PV-INs occurred around P16, the onset of *Pvalb* promoter activation, this experiment cannot discard an earlier involvement of PV-INs. However, it supports a specific role for SST-INs in regulating S1L4 PN wiring.

To dissect whether local SST-IN populations in the cortex control L4 PNs wiring, we ablated SST-INs only in one hemisphere using an adeno-associated viral vector (AAV) containing a Cre-dependent *DTA* (*AAV-EF1*α-*mCherry-flex-DTA*) injected at P2 in S1 of *Sst-Cre* animals. This AAV eliminated SST-INs efficiently, specifically, and reproducibly (Fig. 3D and fig. S8, A to H). We found a ~50% decrease in SST-INs 4 days after injection (P6) (Fig. 3D) and higher reductions thereafter (fig. S8, D to G). AAV-mediated local ablation of SST-INs in S1 increased the number of S1L4 CPNs only in the transduced hemisphere, whereas the contralateral side was unaffected (Fig. 3, E and F). In the injected hemisphere, the number of S1L4 CPNs was similar to that observed in *Sst-Cre;DTA* animals (Fig. 3, B and C). Thus, local S1 SST-INs regulate S1L4 PNs’ wiring by acting at the presynaptic level and not by altering the activity of the PNs’ contralateral targets (*26*, *27*). As with diazepam, the expression of the laminar markers Cux1, Rorβ, and Ctip2 was not altered in the transduced hemisphere or the contralateral non-injected side (fig. S8, I to J).

### Eliminating SST-INs accelerates the onset of PV-IN inhibition

**Fig. 4.**
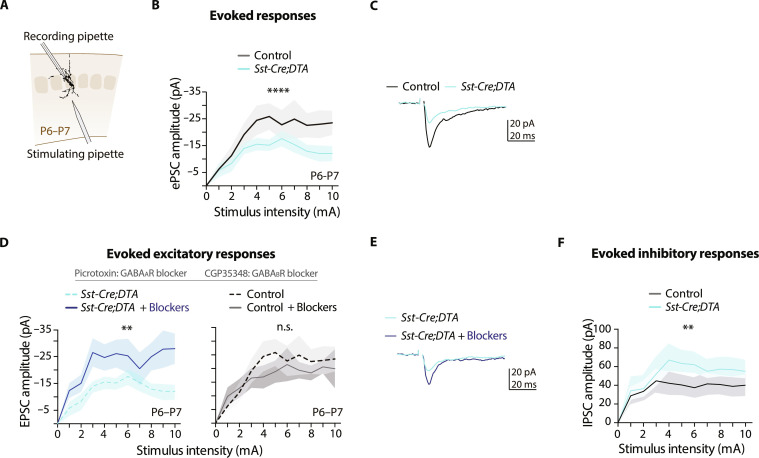
Somatostatin-IN ablation generates a premature increase in inhibition onto S1L4 PNs. (**A** to **C**) Synaptic currents of P6-P7 S1L4 PNs upon deep-layer stimulation (*n* ≥ 9 cells and *n* = 3 mice per condition). (**D**) Effect of GABA_A_ and GABA_B_ receptor blockage on S1L4 responses, which normalize to control values in *Sst-Cre;DTA* mice (right panel same as in Fig. 2J) (*n* ≥ 7 cells and *n* = 3 mice per condition). (**E**) Representative synaptic responses. (**F**) Synaptic currents of S1L4 PNs’ in response to deep-layer stimulation as in (A) to (C) in the presence of NMDA and AMPA/kainite blockers to measure inhibitory events. Increased amplitude of evoked inhibitory responses was observed in *Sst-Cre;DTA* mice (*n* = 17 cells and *n* = 3 mice per condition). Solid lines represent means and shaded area ± SEM. **P < 0.01 and ****P < 0.0001. Two-way ANOVA followed by Šídák’s multiple comparisons test in (B), (D), and (F).

Eliminating SST-INs from the cortex mimicked the effects of diazepam treatment on S1L4 PNs wiring likely because, as reported, the ablation weakens the early functional integration of S1L4 PNs with the thalamus (*12*). To investigate this mechanism, we studied the early wiring of S1L4 PNs in *Sst-Cre;DTA* mice. Immunofluorescence analysis of Vglut2^+^ TC inputs in P6 cortices revealed no significant difference in barrel size or Vglut2 intensity in L4 in *Sst-Cre;DTA* animals compared to *Sst-Cre* mice (fig. S9A and B), although images show a tendency to a minor overspreading of TC inputs onto L2/3 (fig. S9A). Thus, structurally, thalamic innervation was not significantly reduced following SST-IN ablation. Next, we performed whole-cell recordings of S1L4 PNs in P6 acute slices of *Sst-Cre;DTA* mice. These experiments showed slightly reduced intrinsic excitability of *Sst-Cre;DTA* S1L4 PNs and no detectable changes in spontaneous PSCs (fig. S9, C to F). We then evaluated the synaptic integration of S1L4 PNs into local circuitries by stimulating deep layers and measuring evoked synaptic responses (Fig. 4A). In *Sst-Cre;DTA* mice, ePSCs from S1L4 PNs showed significantly smaller amplitudes at different stimulating conditions than controls (Fig. 4, B and C). Notably, bath application of GABAR blockers fully rescued synaptic response to control levels (Fig. 4, D and E, and fig. S9, G and H), demonstrating an overall increase in inhibition upon SST-IN ablations. In regard to synaptic strength, comparisons of the responses of controls and *Sst-Cre;DTA* animals at the lowest stimulation intensity current (1 mA) did not detect significant changes (fig. S10, B and C).

The lack of a GABAR blocker effect in controls but not in *Sst-Cre;DTA* animals suggests abnormal premature inhibition in the mutants. We then evaluated IPSCs at P6-P7. We found that eIPSCs amplitude was increased in *Sst-Cre;DTA* animals compared to controls (Fig. 4F), in agreement with the results obtained with GABAR blockers. There were no detectable changes in spontaneous IPSCs or in the responses at the lowest stimulation current of our protocol (1 mA) (fig. S10, A and D). These results demonstrate that SST-IN ablation leads to unexpected enhanced local inhibition of S1L4 PNs at P6. Such an increase in inhibition functionally disengages S1L4 PNs from excitatory networks and mimics the effect of diazepam. From these findings, together with our previous experiments, we concluded that reductions in the early synaptic integration of S1L4 PNs, either by decreasing excitatory drive (diazepam) or by enhancing inhibition (eliminating SST-INs), are causative of L4 CPN wiring. The experiments showed that, if efficiently recruited by the thalamus and local ipsilateral inputs, then S1L4 PNs later discard interhemispheric connectivity, possibly due to changes in S1L4 PNs’ presynaptic activity and the neuronal assemblies to which they respond.

**Fig. 5.**
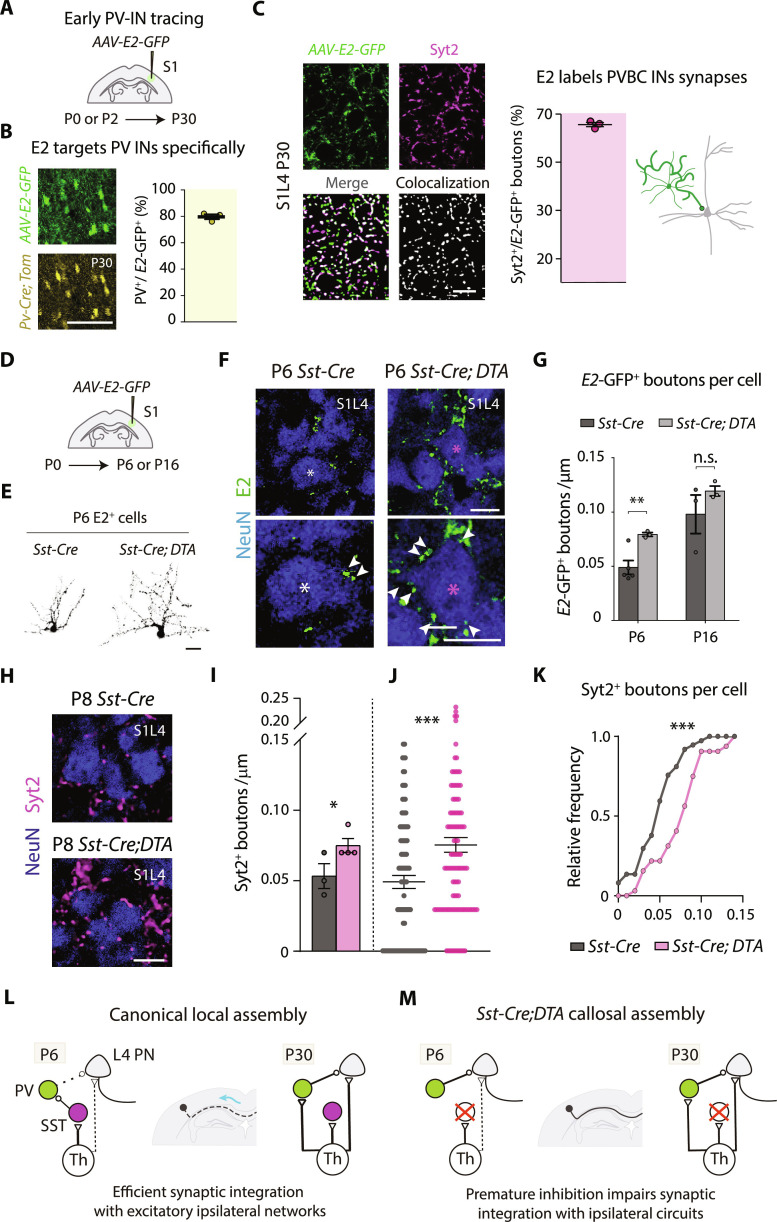
Somatostatin-IN ablation accelerates the onset of PV-IN innervation onto S1L4 PNs. (**A**) Experimental approach in (B) to (C). (**B**) Confocal magnification images in *Pv-Cre;Tom* mice (left) demonstrating the co-labeling between *E2*-GFP^+^ and *Pv*-Tom^+^ cells in S1 at P30 (right) (*n* = 3 mice). (**C**) Confocal images (left) and quantification (right) of colocalization of *E2*-GFP^+^ and PV-specific presynaptic Syt2^+^ boutons in S1L4 at P30 (*n* = 3 mice). (**D**) Scheme of the experiments in (E) to (G). (**E**) Morphology of *E2*-GFP^+^ cells at P6 in *Sst-Cre and Sst-Cre;DTA* mice. (**F**) *E2*-GFP^+^ perisomatic boutons (green) in P6 cortices after *AAV-E2-GFP* transduction at P0. NeuN^+^ (blue). Asterisk (*) indicates the cells magnified in the bottom panels. (**G**) Density of perisomatic *E2*-GFP^+^ clusters per individual S1L4 PN in control and *Sst-Cre;DTA* mutant mice, showing increases at P6 that normalize at P16 (*n* ≥ 3 mice per condition). Dots represent mice-individual values in (B), (C), and (G). (**H**) Confocal images of Syt2^+^ clusters in P8 S1L4 PNs. (**I** to **K**) Quantifications of Syt2^+^ bouton density per S1L4 NeuN^+^ in *Sst-Cre* and *Sst-Cre;DTA* mice at P8. Dots represent mice-individual values in (I), cell-individual values in (J), and cumulative frequency of cell values from (J) in (K) [*n* ≥ 3 mice per condition, *n* ≥ 24 cells per mice, *n* = 98 cells (*Sst-Cre*), and *n* = 111 cells (*Sst-Cre;DTA*)]. (**L** to **M**) Model. In (L), canonical scenario in which efficient recruitment of thalamic (Th) and local inputs onto S1L4 PNs generates early synaptic integration with ipsilateral circuits and callosal retraction. In (M), ablation of SST-INs produces a paradoxical increase in PV-mediated inhibition onto S1L4 neurons, disengaging them from the ipsilateral circuit and increasing adult callosal connectivity. Data are means ± SEM. **P < 0.01 and ***P < 0.001. Unpaired *t* test in (G), (I), and (J) and Kolmogorov-Smirnov in (K). Scale bars, 100 μm (B), 20 μm (E), and 10 μm [(C), (F), and (H)].

The source of enhanced inhibition in P6 *Sst-Cre;DTA* cortex remained unsolved. The developmental onset of inhibition is specific to IN-PN motifs (*3*, *36*). S1L4 PNs receive most inhibitory inputs from PV-INs but only after P6 to P7 (*14*). On the other hand, previous work has shown that SST-INs transiently innervate PV-INs during the first postnatal week to mediate the maturation of cortical circuits (*11*). Therefore, we hypothesized that PV-INs could be deregulated in *Sst-Cre;DTA* mice. In line with this, we observed brighter PV immunoreactivity (*37*, *38*) in *Sst-Cre;DTA* animals compared to *Sst-Cre* controls (fig. S11, A and B). Therefore, we investigated whether the increased synaptic inhibition received by S1L4 PNs in P6 *Sst-Cre;DTA* animals was due to PV-IN dysregulation. Because the late expression of the *Pvalb* promoter precluded its use to label early PV-INs, we resourced to the recently identified enhancer element “*E2*” to illuminate developing PV-INs and their synaptic inputs (*AAV-E2-GFP*) (Fig. 5, A to G) (*39*). First, we confirmed the specificity of the targeting approach. Injection of this AAV into S1 of *Pv-Cre;Tom* mice at P2 demonstrated that ~80% of *E2*-GFP^+^ cells were *Pv*-Tom^+^ at P30 (Fig. 5B and fig. S11, C and D). Moreover, when examining GFP^+^ puncta, they were in close apposition to gephyrin (fig. S11E), and almost 70% of *E2*-GFP^+^ clusters in S1L4 colocalized with synaptotagmin 2 (Syt2^+^), a reliable marker of PV-basket synapses (Fig. 5C and fig. S11, E and L). Once we confirmed *E2* selectivity, we analyzed early PV-IN inputs onto S1L4 PNs upon SST-IN ablation. *AAV*-*E2-GFP* vector was injected at P0 into S1 of *Sst-Cre;DTA* and *Sst-Cre* mice. The injections demonstrated high expression efficiencies and comparable spreading and specificity of the AAV in all animals (fig. S11, F to K). Quantifications of *E2*-GFP^+^ clusters at P6 revealed an increased density of perisomatic PV-IN contacts onto S1L4 PNs in *Sst-Cre;DTA* animals compared to *Sst-Cre* controls (Fig. 5, D to G). By contrast, there were no differences in PV-IN contacts onto S1L4 PNs at P16 (Fig. 5G and fig. S11M).

Further analysis at P8, the onset of Syt2^+^ clusters exponential growth in upper layers (*3*), confirmed accelerated PV-IN innervation onto S1L4 PNs and increased number of Syt2^+^ boutons per cell in *Sst-Cre;DTA* mice (Fig. 5, H to K). The same finding was confirmed in the local (AAV-mediated) elimination of SST-INs, as Syt2 staining revealed a specific increase in PV-IN to S1L4 PN innervation at P12 that was normalized to control values at later stages (fig. S12, A to D). Thus, the elimination of SST-INs leads to premature PV-IN innervation onto S1L4 PNs (Fig. 5, L and M). This premature PV-IN innervation likely contributes to the observed increase in inhibition (Fig. 4F) and, hence, to the later rewiring of S1L4 PNs.

### Callosal axons from S1L4 CPNs in SST-IN mutants are functionally active

**Fig. 6.**
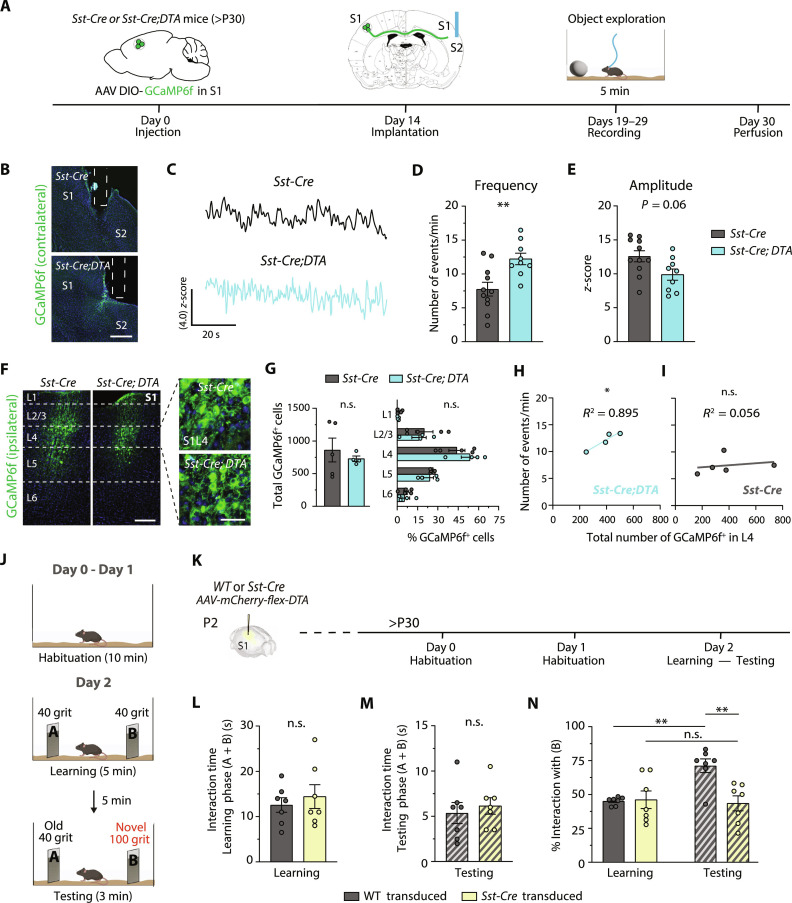
Calcium transients of the induced S1L4 interhemispheric axons and whisker-sensory deficits upon SST-IN elimination. (**A**) Young adult mice were injected with an AAV expressing the calcium sensor GCaMP6f (*AAV*-*CaMKII-GCaMP6f-WPRE-SV40*) in S1 of the right hemisphere. After, we implanted an optical ferrule in the contralateral hemisphere to visualize the activity of interhemispheric axons using fiber photometry during 5-min object exploration sessions. (**B**) Confocal images of the contralateral area in control and mutant mice. Dashed line indicates the positioning of the fiber-optic cannula. (**C**) Examples of calcium traces. (**D**) Frequency of calcium transients. (**E**) Mean amplitude. (D) and (E) Mean values per recording session (≥2 sessions per mouse). (**F** and **G**) Confocal images (F) and quantification and distribution per layer (G) of the AAV-transduced (GCaMP6f^+^) cells. (**H** and **I**) Plot of GCaMP6f^+^ L4 cell number versus mean transient frequency during the task. Pearson’s correlation analysis shows a significant correlation in *Sst-Cre;DTA* mice (H) but not in controls (I). (A) to (I) (*n* = 5 *Sst-Cre* mice and *n* = 4 *Sst-Cre;DTA* mice). (**J**) Behavioral paradigm for whisker-based sensory analysis. (**K**) Injections in *Sst-Cre* and WT mice with the *AAV-EF1*α*-mCherry-flex-DTA* vector at P2 followed by behavioral assessment at P30 and onward. (**L** to **N**) Time spent interacting with textures A and B during the learning (L) and testing (M) phases. (N) Time percentage interacting with the B texture in the learning (A and B textures are equal) and testing phase (B representing a novel texture) (*n* = 7 animals per condition). Data are means ± SEM. *P < 0.05 and **P < 0.01. Nested [(D) and (E)] and unpaired [(G), left, (L), and (M)] *t* tests, one-way (N) and two-way [(G), right] ANOVAs followed by Šídák’s multiple comparisons tests, and Pearson’s correlations [(H) and (I)]. Scale bars, 200 μm [(B) and (F), left] and 20 μm [(F), right]. WT, wild type.

Our previous studies in anesthetized mice demonstrated that noncanonical L4 CPNs from the adult visual cortex provide functional interhemispheric connectivity (*8*). Here, we assessed the in vivo activity of noncanonical S1L4 callosal axons in *Sst-Cre;DTA* mutants during behavior. We injected *Sst-Cre;DTA* and control (*Sst-Cre*) adult mice (7 to 12 weeks) with an AAV expressing the calcium sensor GCaMP6f (*AAV CaMKII-GCaMP6f-WPRE-SV40*) into S1L4 of the right hemisphere. Two weeks later, we implanted an optical ferrule in the contralateral (left) hemisphere to record the activity of the interhemispheric axons from the transduced PNs using fiber photometry (Fig. 6A). The ferrule was located in the somatosensory area at the S1/S2 border, the branching site of induced S1L4 callosal axons [see Fig. 1F and (*8*)]. The recordings in the contralateral cortex were performed in freely moving animals while they executed a 5-min object exploration task (Fig. 6B). We analyzed the frequency and average amplitude of the recorded calcium transients (Fig. 6, C to E). The results showed higher frequency in *Sst-Cre;DTA* mice than controls (Fig. 6, C and D), indicating increased interhemispheric activity during object exploration. The mean amplitude showed no statistical differences (Fig. 6, C and E), although there was a tendency to decrease in *Sst-Cre;DTA* mice, suggesting that interhemispheric axons in *Sst-Cre;DTA* mice spike more often but perhaps with less synchrony. The distance traveled and the duration of object exploration were similar between groups (fig. S13, A to F), indicating that the change in calcium activity was not due to alterations in locomotion or exploration. Post hoc analysis quantifying GCaMP6f^+^ (AAV-transduced) cells in implanted brains showed comparable transduction efficiencies and similar layer distributions of GCaMP6f^+^ cells between *Sst-Cre;DTA* and control mice. In both groups, most of the transduced cells were located in L4, and there were equivalent numbers of off-targeted cells in L2/3 and L5 (Fig. 6, F and G). Thus, the increase in activity was not due to differences in the number or layer distribution of the transduced cells expressing the sensor. Notably, plotting the mean frequency value of each mouse against the number of GCaMP6f^+^ L4 cells demonstrated a linear correlation, with the frequency increasing proportionally with the number of transduced L4 PNs in *Sst-Cre;DTA* (Fig. 6H). There was no correlation in control mice (Fig. 6I), supporting that L4 PNs do not contribute to interhemispheric activity. Conversely, in control animals, the frequency of calcium transients correlated with the number of GCaMP6f^+^ L2/3 and L5 PNs (fig. S13, G and H) as expected from the canonical circuit (*8*). During the experiment, calcium activity correlated with the animals interacting with the object (fig. S13I), indicative of whisker-derived activity. Overall, these results demonstrate that interhemispheric axons from S1L4 CPNs of *Sst-Cre;DTA* mice are active during object exploration.

### Elimination of local SST-INs disrupts whisker-mediated sensory perception

Last, to test the consequences of SST-INs ablation and augmented S1L4 interhemispheric connectivity on fine sensory processing, we assessed the animals’ ability to discriminate textures with the whiskers (*40*–*42*). For this, we used an established protocol whereby animals can explore two objects (panels) with equal textures (sandpaper) during a learning period. Afterward, the coarseness of the texture is changed for only one of the objects. The object with a new texture is recognized as novel, and mice spend more time investigating it. The ability of mice to discriminate textures is therefore assessed by a preferential interaction with the new texture (Fig. 6J). We performed this study in mice with local cortical ablations of SST-INs in one hemisphere, i.e., *Sst-Cre* heterozygous animals unilaterally injected with the *AAV-flex-DTA* vector (Fig. 6K). This ensures that the analysis evaluates only cortical functions and the effect of the extra S1L4 CPNs on sensory integration. We also injected WT mice with the same AAV construct to control for the impact of the craniotomy and AAV injection. In both the learning and testing phases, the time animals spent exploring the objects was identical between the two groups, thus proving comparable exploratory behavior and interest for novel objects (Fig. 6, L and M). In the learning phase, control and *Sst-Cre*;*AAV-DTA* animals showed no preference for any of the two equal objects/textures. Then, during the testing phase, control animals preferentially explored the novel texture (Fig. 6N) and performed equally well compared to non-injected WT mice (fig. S13J), thus proving that the injections did not affect whisker-mediated discrimination. By contrast, *Sst-Cre;AAV-DTA* animals proved incapable of recognizing the novel texture (Fig. 6N). Because SST-IN ablation occurs in only one hemisphere, the data support the conclusion that interhemispheric activity from S1L4 CPNs disrupts texture discrimination. Overall, our results demonstrate that ipsilateral local SST-INs ablation is sufficient to impair bilateral sensory integration through the adverse effects of excessive S1L4 CPNs on sensory perception.

In conclusion, we show that decreasing the excitatory/inhibitory (E/I) input balance onto S1L4 PNs’ during a P1 to P7 critical period promotes their maturation as CPNs. We further demonstrate that local SST-INs determine the canonical local wiring of S1L4 PNs. SST-INs developmentally delay PV-IN–mediated inhibition of differentiating S1L4 PNs, allowing the PNs integration into early ipsilateral excitatory network assemblies, which is necessary to induce the shedding of S1L4 PNs interhemispheric axons, and thus prospectively preventing CPN connectivity. Overall, our data indicate that maturation of the barrel cortex requires the trimming of excessive contralateral outputs from L4 for fine processing of sensory information and supporting behaviors such as texture discrimination.

## DISCUSSION

### SST-INs regulate barrel assembly and local S1L4 PNs’ wiring

We herein demonstrate that cortical SST-INs, early recipients of thalamic inputs (*11*, *12*), determine the canonical local wiring of S1L4 PNs. SST-INs act as a “developmental brake” for PV-IN’s innervation, crucial for the correct elimination of callosal axons from S1L4 PNs. The effect is mediated presynaptically by ipsilateral local SST-PV-IN networks, as shown by the ablations performed only in one cortical hemisphere. Our findings provide an unreported function for the developmental SST-PV-IN interaction described by (*11*), expand previous interpretations of the importance of transient connectivity (*12*), and overall, reinforce the idea of SST-INs as developmental “hub” neurons that coordinately regulate several aspects of S1 barrel connectivity. The results demonstrate the role of INs and presynaptic inhibition in sculpting excitatory axonal projections.

Our data align with the increasing body of studies evidencing a coordinated model of canonical wiring (Fig. 5L). Initially, the thalamus dictates the activity of deep-layer SST-INs (*11*, *12*), which, in turn, establish transient connections with both PV-INs (*11*) and L4 PNs (*12*). As development proceeds, TC inputs gradually migrate from the subplate onto S1L4 while PNs extend their transitory callosal projections (*8*). We show here that, during this process, SST-INs act as a brake on PV-INs inputs onto S1L4 PNs. By the time developmental callosal axons reach homotopic territories, thalamic activity has already engaged S1L4 PNs into whisker-active assemblies (*29*, *33*). The resulting activity promotes the shedding of developmental callosal axons. Whether this process involves synaptic plasticity or other mechanisms, such as changes in the intrinsic molecular composition of the neuron or the axon or changes in intrinsic electrophysiological properties, remains to be studied. Last, with S1L4 PNs acquiring the input connectivity that will establish their adult, local-only connectivity, SST-INs retract their connections with PV-INs, coinciding with the onset of active whisking (*11*). PV-INs can then innervate S1L4 PNs, thus increasing whisker selectivity (*14*). SST-INs, therefore, control the building of the specialized microcircuit in charge of somatosensory perception.

Diazepam treatment affects the excitatory component of S1 canonical assembly, mainly the establishment of excitatory thalamic inputs onto S1L4 PNs, which SST-INs developmentally regulate. How precisely diazepam creates this remodeling of early excitatory networks is unclear. Predicting the effects of increasing GABAR signaling systemically is challenging, as numerous cellular players and types of plasticity could be affected. With our analyses, we cannot support or discard any specific direct mechanism of action. There are many possibilities. To name a few, we cannot discard the direct dampening of thalamic activity, nor can we fully discard effects on the activity of SST-INs, which, in turn, would indirectly impair the development of thalamic inputs onto S1L4 PNs. Given that our main focus was the dissection of callosal wiring, a detailed mechanism was out of the scope of the present article. However, the experiments pave the way for future studies.

Genetic depletions of SST-INs univocally demonstrate the essential role of presynaptic INs for the canonical wiring of S1L4 PNs. While previous works demonstrated that PNs’ activity and excitability influence callosal circuits (*8*, *26*, *27*, *43*–*46*), the role of inhibition has only recently been explored. An interesting recent study by (*5*) showed that disrupting GABA_A_Rs expression in S1L2/3 CPNs enhanced their interhemispheric synchrony and increased contralateral branching. Thus, eliminating the inhibitory component and favoring the recruitment of S1L2/3 PNs into an active interhemispheric ensemble promotes contralateral terminal branching. This is the opposite of our observations in L4 and suggests a context-dependent effect of developmental inhibition. Our data show that early inhibition promotes CPN connectivity, while the robust functional integration of differentiating S1L4 PNs within nascent ipsilateral excitatory circuits leads to the elimination of their developmental callosal axons. Thus, inhibition can favor or eliminate CPN wiring, possibly depending on the developmental time of inhibition, the subtype of PNs, their differentiation status, and the developmental stage of their axons. Considering their remarkable diversity and the fine regulation of their synaptic partners (*3*, *15*, *16*), INs appear ideal for generating circuit diversity at a functional and structural level.

Another interesting point is that we found evidence of functional PV-INs before they express detectable levels of the PV marker. While PV-IN activity has been assumed to be limited until they express PV protein, recent studies (*13*, *47*) have shown that PV-INs are active and regulate population dynamics at early postnatal stages. Moreover, the increased inhibition of S1L4 neurons observed in *Sst-Cre;DTA* animals agrees with previous studies showing that preventing action potential-dependent neurotransmitter release from SST-INs at P4 to P6 results in reduced thalamic responses and a compensatory increase in local L4 GABAergic inhibition (*12*).

### Early plasticity mediates alternative adult projection patterns in canonical and noncanonical scenarios

Overall, our data support a wiring model in which cortical PNs gradually engage in active circuits while discarding potential connectivity. This allows the emergence of circuit diversity without the need for complex preprogrammed molecular instructions hard-wired in young PNs. INs, because they can functionally subtract PNs from active ensembles, are perfect regulators of this process. Fiber photometry shows that the induced noncanonical S1L4 CPNs are functionally active, which, together with the behavioral analysis, indicates that the excessive S1L4 CPN connectivity causes deficits in whisker-mediated sensory perception. In regard to the molecular changes that S1L4 PNs undergo during this process, it is a question that will require further studies. It is becoming increasingly evident that the initial identity of young cortical neurons is only partially committed. At the very early stages of neuronal differentiation, only two broad main cortical subtypes, subcortical and intracortical projection PNs, are molecularly distinguished (*6*). Cortical neurons gradually acquire their adult subclass-specific identities during postmitotic differentiation (*6*, *8*, *9*, *43*, *48*–*51*). Furthermore, we and others showed that the default wiring program of upper-layer PNs is callosal. Notably, developmental exuberant callosal axons reflect a shared undifferentiated stage of young neurons at the beginning of their maturation program (*6*, *8*). An early neuronal CPN identity that is gradually modified by activity-dependent mechanisms agrees with the absence of a unique molecular fingerprint associated with callosal or local wiring and the fact that several different identities mark CPNs (*52*, *53*). Our previous studies also concluded that the same transcriptional fingerprint can give rise to alternative mature projection patterns in canonical and noncanonical situations (*50*). The data herein reported further support that local or interhemispheric wiring depends on the PNs’ early molecular programs combined with the activity of early presynaptic inputs and potential targets. Hence, understanding the developmental context of PNs is essential to predict wiring patterns from the identity of mature neurons. In this regard, we have no evidence supporting that CPN wiring requires changes in subclass-specific programs. Still, our investigations did not conduct in-depth molecular characterization, and we cannot discard that INs’ inputs regulate aspects of the molecular identity of PNs. Last, it is noteworthy that our findings affect the current understanding of NDDs, as abnormal cortical E/I balance is a frequent hallmark of NDDs (*54*–*57*). Our research therefore adds to a growing body of evidence explaining why certain disorders with shared symptomatology, such as schizophrenia or autism spectrum disorder, can arise from genetic mutations affecting such distinct neuronal populations as INs and PNs.

## MATERIALS AND METHODS

### Mice

*Ror*β*-IRES2-Cre-D* *Ror*β*^Cre/+^*; Jackson Laboratories (JAX), no. 023526 (*20*), *Sst-IRES-Cre* (*Sst^Cre/+^*; JAX, no. 028864) (*58*), *Pv-IRES-Cre* (*PV^Cre/+^*; JAX, no. 017320) (*59*), *R26:lacZbpA(flox)DTA* (*DTA^f/+^*) (*60*), *Ai14(RCL-TdT)-D* (*Tomato^f/+^*; JAX, no. 007914) (*61*), and *R26-tm1(CAG-EGFP)Fsh* (*GFP^f/+^*; JAX, no. 032038) (*62*) were maintained in a C57BL/6 background (Charles River Laboratories). The morning of the appearance of a vaginal plug was defined as E0.5. Animals were maintained under standard, temperature-controlled conditions on a 12-hour:-12-hour light/dark cycle. Water and food were provided ad libitum. Animals were housed and maintained following the guidelines from the European Union Council Directive (86/609/ European Economic Community). All the procedures for handling and sacrificing animals followed the European Commission guidelines (2010/63/EU). All animal procedures were approved by the Consejo Superior de Investigaciones Científicas (CSIC) and the Community of Madrid Ethics Committees on Animal Experimentation in compliance with national and European legislation (PROEX 118-14; 233/16; 124-17; 234-16; 123-17; 065/19; 162.6/23; 2020/VSC/PEA/0227).

### Diazepam treatment

Diazepam solution for injection of 5 mg/ml (*Roche*, ref. 718796.7) was diluted with physiological saline (0.9% NaCl) to obtain a final dose of 10 mg/kg mice weight. The treatment was administered intraperitoneally once a day for the duration of different postnatal windows. Because diazepam can hinder pups’ feeding, the weight of the animals was monitored daily. If a pup’s weight was significantly lower than that of its control littermates, then hand-forced feeding was used to supplement its diet (*63*).

### CTB injections for retrograde labeling

Retrograde labeling from the CC was performed by injecting the CTB conjugated to Alexa Fluor 555 or 647 (Thermo Fisher Scientific, refs. C-34776 and C-34778) into the CC of one hemisphere. As reported in (*8*), stereotaxic coordinates (in millimeters) in the anteroposterior (AP), mediolateral (ML), and dorsoventral (DV) axes were determined from Bregma. For the analysis of somatosensory CPNs, coordinates and CTB volumes were as follows (in millimeters from Bregma): for P10 (−1.1 AP, +0.7 ML, and −1.8 DV; 300 nl), for P14 (−1.4 AP, +0.7 ML, and −1.9 DV; 460 nl), and for P21 to adult (−1.4 AP, +0.7 ML, and −2 DV; 575 nl). For the analysis of visual and auditory CPNs, coordinates were as follows: for P21 to adult (−2.4 AP, +0.7 ML, and −1.5 DV; 575 nl).

Animals were anesthetized during the surgical procedure with isoflurane/oxygen and placed on a stereotaxic apparatus (Harvard Apparatus) with a mouse and a neonatal adapter (Stoelting). CTB, diluted at 0.5% in phosphate-buffered saline (PBS), was injected with a Drummond Nanoject II Auto-Nanoliter Injector using 30-mm pulled glass micropipettes (Drummond Scientific, refs. 3000205 A and 3000203 G/X). Injections were made at 23 nl per injection pulse with a maximum frequency of one pulse per second (23 nl/s) to minimize damage until the desired total volume was achieved. We allowed at least 48 hours after injection before perfusing the animals for analysis.

### AAV production and intracranial injections

Plasmids *pAAV-EF1*α*-mCherry-flex-DTA* (Addgene, no. 58536) (*64*) and *pAAV-S5E2-GFP-fGFP* (Addgene, no. 135631) (*39*) were used to create AAV for gene delivery. AAV serotype 8 was chosen because it shows strong neuronal tropism and high transgene expression in the brain (*65*, *66*). The Viral Vector Unit of the Universidad Autónoma de Barcelona and the Neurotropic Vectors Unit of the Instituto de Neurociencias de Alicante encapsulated the vectors. Briefly, they performed a triple transfection of HEK-293 cells with (i) the abovementioned plasmids, (ii) a rep-cap vector producing the viral proteins, and (iii) a helper plasmid containing adenovirus genes required for replication (E4, E2a, and VA). The resulting titers were 1.05 × 10^13^ genome copy (gc)/ml (*pAAV-EF1*α*-mCherry-flex-DTA*) and 7.52 × 10^13^ gc/ml (*pAAV-S5E2-GFP-fGFP*), respectively. For S1 injections at P0 and P2, stereotaxic coordinates were adjusted using the atlas of Paxinos (*67*): −1 mm AP, +1.6 mm ML, and −0.3 to −0.5 mm DV, relative to Bregma. AAVs were diluted 1:2 in 0.9% NaCl solution containing FastGreen, and 250 nl was injected.

### Plasmids and IUE

Plasmids used were *CAG-GFP* (Addgene, no. 11150) (*68*), *CALNL-DsRedExpress* (cloned from the *pCAGGs-DsRedExpress*, provided by C. Lohmann from the Netherlands Institute for Neuroscience), and the *pCALNL-GFP*, containing a flox-stop-flox sequence for CRE-dependent expression of green fluorescent protein (GFP; a gift from C. Cepko from Harvard University; Addgene, no. 13770) (*68*). IUE was performed as previously described (*69*). Briefly, pregnant mice were anesthetized with isoflurane/oxygen, and a solution containing the specified plasmids at a concentration of 1 μg/μl was injected into the embryo’s lateral ventricle using a 30-mm pulled glass micropipette before five voltage pulses (36 mV, 50 ms) were applied using external paddles oriented to target S1.

### Perfusion and immunohistochemistry

Mice were deeply anesthetized by intraperitoneal injection of xylazine (Xilagesic, Calier) and ketamine (Imalgene Merial Laboratorios) solution. Transcardial perfusion was performed with a 10% formalin solution (Sigma-Aldrich, ref. HT501128-4 L). Brains were postfixed overnight in formalin at 4°C and cryoprotected by immersion in 30% sucrose (Merck, no. S0389) for 48 hours. For the analysis of vesicular and synaptic proteins, mice were perfused with PBS followed by 4% paraformaldehyde (PFA) (Merck, ref. 818715) solution in PBS. Brains were kept in 4% PFA for 3 hours and transferred to sucrose 15% for 12 hours and then to 30% sucrose for 48 hours. The tissue was then frozen in Tissue-Tek O.C.T. Compound (Sakura Tissue-Tek, ref. 4583). Free-floating sections (50 μm) were used for immunostaining. Floating sections were immunostained with antibodies during incubation times ranging from 24 to 48 hours at 4°C in a solution composed of PBS with 0.5% Triton X-100 (Sigma-Aldrich, T9284) (PBS-T) and 5% fetal bovine serum (FBS). Then, sections were washed and incubated with the secondary antibodies diluted in PBS-T and 3% FBS. For the analysis of molecular markers, 50-μm sections were attached onto superfrost adhesion slides (Epredia J1800AMNZ) and underwent a heat-induced antigen retrieval protocol (citrate buffer, pH 6.0; Sigma-Aldrich, C9999) before immunostaining with Cux1, Rorβ, and Ctip2 antibodies in 5% FBS in PBS-T at 4°C during 24 hours. For GCaMP6f amplification, we anesthetized the mice using isoflurane and then perfused transcardially with 10 ml of saline, and their brains were quickly extracted and incubated in 4% PFA overnight. After 1 hour of washing in 0.3 M glycine in PBS, 50-μm slices were prepared using a Leica VT1000S vibratome (Leica Biosystems). Unless indicated otherwise, slices were permeabilized for 30 min in PBS-T before being incubated overnight at 4°C with the anti-GFP polyclonal antibody, Alexa Fluor 488 (no. A21311) diluted in PBS-T. The slices were washed in PBS for 1 hour, and, then, Hoechst counterstain was applied [Hoechst 33342 at 1:1000 for 30 min in PBS at room temperature (RT)] before mounting the slice using fluor mount (Sigma-Aldrich).

### Antibodies

Antibodies used are as follows: rabbit anti-GFP (1:500; Thermo Fisher Scientific, Invitrogen, A11122); rabbit anti-GFP polyclonal antibody, Alexa Fluor 488 (1:500; Thermo Fisher Scientific, no. A21311); rabbit anti-Cux1 (1:500; Santa Cruz Biotechnology, sc-13024 X); mouse anti-human Rorβ (1:300; Perseus Proteomics, PP-N7927-00); rabbit anti-Ctip2 (1:250; Abcam, ab240636); guinea-pig anti-Vglut1 (1:500; Merck Millipore, AB5905); mouse anti-NeuN (1:500; Chemicon, MAB377; clone A60); guinea pig anti-Vglut2 (1:1000; Sigma-Aldrich, Merck Millipore, AB2251-I); mouse anti-gephyrin (1:250; Synaptic Systems, no. 317005); mouse anti–synaptotagmin-2 (1:250; ZFIN, no. ZDB-ATB-081002-25); mouse monoclonal anti-PV (1:500; Sigma-Aldrich, Merck Millipore, no. p3088); goat anti-mouse Alexa Fluor 405 (1:200; Thermo Fisher Scientific, no. A-31553); goat anti-mouse Alexa Fluor 488 (1:500; Life Technologies, no. A-11029); goat anti-rabbit Alexa Fluor 488 (1:500; Life Technologies, no. A-11034); goat anti-rabbit Alexa Fluor 647 (1:500; Thermo Fisher Scientific, no. A-A21245); goat anti-mouse Alexa Fluor 647 (1:500; Thermo Fisher Scientific, no. A21236); and goat anti–guinea pig Alexa Fluor 647 (1:500; Thermo Fisher Scientific, no. A21450).

### Imaging and analysis

Images of brain sections were reconstructed using either the Leica DMI6000B fluorescence microscope or the inverted Leica TCS-SP8 confocal. For all the quantitative analysis, we used the Leica TCS-SP5 or TCS-SP8 confocal microscopes. Up to 50-μm optical *z*-sections were obtained by taking 3.5-μm serial sections with LAS AF v1.8 software (Leica). Images were acquired maintaining the same laser power, photomultiplier gain, pinhole, and detection filter settings among conditions. For CTB, fluorescence, and immunostaining quantifications, mosaic images were obtained with the 20× objective at 512 × 512 resolution (8 bits). The tilescan tool of the Leica LAS AF software was used to reconstruct the images. For the quantification of vesicular proteins, single *z*-stacks were acquired with the 100× oil immersion objective at 1024 × 1024 resolution (8 bits). Images were analyzed using ImageJ (Fiji) (*70*).

Quantification of CTB^+^ cells was performed across single confocal plane images from *z*-stacks using 4′,6-diamidino-2-phenylindole (DAPI) and CTB staining. In each layer, first, a minimum of 50 randomly selected nuclei DAPI^+^ are demarked, and, subsequently, CTB filling is analyzed in each of them. For each cell, the planes on top and under in the *z*-stacks were analyzed to ensure that the fluorescent signal surrounds the DAPI nuclei and that the neuron is filled with CTB. Data are provided as the percentage of CTB^+^ neurons out of the selected DAPI^+^ cells (minimum of 50 cells per layer). Quantifications were performed in the hemisphere contralateral to the site of injection. This ensures that all labeled neurons have at least one interhemispheric projection in the contralateral territory, regardless of the growth or injury status. To delimit the equivalent areas in the developing brain, we used the Developing Mouse Reference Atlas from Allen Brain Atlas (http://atlas.brain-map.org/). Neuronal markers and infection efficiencies were measured in the same manner.

For the analysis of the IUE, measures of the ipsilateral somas and the contralateral innervation were performed as previously reported (*8*). To check for comparable IUE efficiency, we quantified the total number and distribution of electroporated neurons in a semiautomated manner using an ImageJ custom macro already used in (https://doi.org/10.5281/zenodo.10423646) (*21*). Briefly, the entire electroporated region was outlined, and cortical layers were separated on the basis of their distinct cell densities. The threshold was set to identify neuronal somas, and the cell numbers in each layer were obtained using the script. The selected regions of interest were then manually checked. Quantification of the *Sst*-Tomato^+^, *Sst*-GFP^+^, and *Pv*-Tomato^+^ cells, as well as counting of PV immunopositive cells, was done with the same custom macro in manually drawn regions of interest (ROIs) (S1 or S2). For measuring innervation, manually selected ROIs were drawn around the contralateral axons. The threshold was adjusted above the noise (making a binary image), and the integrated density was measured. The values in contralateral ROIs were normalized to the ones in the ipsilateral area of the same coronal section.

Quantification of the fluorescence of PV immunostaining was done with a self-written macro (https://doi.org/10.5281/zenodo.10423646). The user manually limits S1 and the layers within the area. The script detects the positive cells and measures their fluorescence and the background of each laminae. Those data are then used to quantify the corrected total cell fluorescence (CTCF). CTCF is calculated by subtracting from the integrated density of each cell, the product of its area and mean background fluorescence.

Analysis of IN synapses and PV-IN cells: To illuminate developing PV^+^ cells and their synaptic inputs in *Sst-Cre;DTA* animals, we resourced to a recently identified enhancer element, *E2*, that is active in differentiating PV-INs (*39*). For vesicular markers (Syt2 and gephyrin), analysis was performed using a self-written macro (https://doi.org/10.5281/zenodo.10423646). Briefly, IUE or NeuN^+^ cells were outlined on individual confocal Z planes, and the puncta apposed (Syt2) or inner (gephyrin) to the outlined somas within a 0.1 μm width band were automatically quantified. Overlap between *E2*-GFP^+^ fluorescence and Syt2 staining was analyzed with a self-written macro (https://doi.org/10.5281/zenodo.10423646) on the basis of the strategy explained in (*71*). Clusters were defined using the log3D plug-in. The binary image was used to count the number of clusters (>0.04 μm), with the results incorporated into the ROI manager. The threshold image of Syt2 was used to show the *E2*-GFP^+^ clusters saved on the ROI manager and the integrated density measured. An integrated density value of >0 was considered double positive.

### Ex vivo electrophysiological recordings

Mice (P6-P7) were euthanized, and their brains were quickly and carefully removed and placed in ice-cold artificial cerebrospinal fluid (aCSF) containing 124 mM NaCl, 2.69 mM KCl, 1.25 mM KH_2_PO_4_, 2 mM MgSO_4_, 26 mM NaHCO_3_, 2 mM CaCl_2_, and 10 mM glucose and were gassed with carbogen (95% O_2_/5% CO_2_,180; pH 7.3). Slices (300-μm thick) were obtained with a vibratome (Leica Vibratome VT1200S) and incubated (> 1 hour) at RT in aCSF continuously gassed. Slices were then transferred to an immersion recording chamber superfused at 2 ml/min with gassed aCSF and visualized under an Olympus BX50WI microscope (Olympus Corporation) coupled with a 40× water immersion lens and infrared–differential interference contrast optics.

Electrophysiological recordings from neurons located in L4 of the somatosensory barrel were made using whole-cell patch-clamp technique and borosilicate capillaries (3-10 megaohms) filled either with an intracellular solution containing 135 mM K-gluconate, 10 mM KCl, 10 mM Hepes, 1 mM MgCl_2_, and 2 mM adenosine 5′-triphosphate (ATP)–Na_2_ (pH 7.3 adjusted with KOH) or with a Cs-based intracellular solution containing 127 mM CsMeSO_3_, 2 mM CsCl, 10 mM Hepes, 5 mM EGTA, 4 mM QX-314Br, and 4 mM MgATP (pH 7.3 adjusted with CsOH). Membrane potential (millivolts), membrane capacitance (picofarads), and membrane resistance (megaohms) were measured at the onset and end of recordings. For action potential discharges, current-clamp recordings were performed (40-pA injecting current steps) in neurons patched with the K-based intracellular solution. All rest recordings were performed in voltage-clamp conditions. To study PSCs, neurons were held at a holding potential (V_h_) of −70 mV; IPSCs were recorded by voltage-clamping the cell near the equilibrium potential for glutamate (EGlut; starting at −70 mV and gradually changed in 10-mV steps to 0 mV). For IPSCs recordings, V_h_ was set to 0 mV. In addition, the glutamatergic blockers, AP5 (50 μM) and CNQX [Cyanquixaline (6-cyano-7-nitroquixaline-2,3-dione)] (20 μM) were added to the aCSF to ensure the isolation of inhibitory responses. In a subset of experiments, picrotoxin (50 μM) was added to the aCSF, and IPSCs were abolished, demonstrating the GABAergic nature of recorded responses.

Recordings were obtained with PC-ONE amplifiers (Dagan Corporation). Series and input resistances were monitored, and recordings with access resistance >20% during the experiment were rejected. Signals were fed to a Pentium-based PC through a DigiData 1440 interface board (Axon Instruments). Signals were filtered at 1 kHz and acquired at 10-kHz sampling rate. The pCLAMP 10.7 software (Axon Instruments) was used for stimulus generation, data display, acquisition, and storage. All the experiments were performed at RT.

Spontaneous postsynaptic currents were recorded for 10 min. Synaptic stimulation was achieved using theta capillaries (2- to 5-μm tip diameter) filled with aCSF. The stimulation electrodes were placed in L5 of the somatosensory cortex. Single pulses of increasing intensity (1 to 10 mA) were delivered to measured dose-response curves. For evoked synaptic currents, paired pulses (250-μs duration, 75-ms interval) were continuously delivered at 0.33 Hz by stimulator S-900 (Dagan 205 Corporation). To isolate excitatory postsynaptic currents, picrotoxin (50 μM) and CGP55845 (5 μM) were added to the aCSF. For the analysis of electrical responses at 1 mA, 5 to 10 consecutive sweeps were considered, and synaptic efficacy (average of all responses including failures), synaptic potency (amplitude of responses), and synaptic failure (ratio of responses from the total of stimuli applied) were measured as previously described (*34*).

### Novel texture discrimination test

To assess somatosensory function, we performed a novel texture discrimination test (*40*–*42*). The experiment is divided into three phases: habituation, learning, and testing. In the first phase, over two consecutive days, mice are habituated to the testing arena, a white opaque uncovered box (35 cm by 35 cm by 35 cm) with 2 cm of cage bedding, for 10 min each day. The learning and testing phases are carried out on the third day and test the ability to recognize a novel texture. For this, each mouse is introduced for 5 min in the testing arena and exposed to two identical rectangular panels of methacrylate (4 cm by 15 cm), which surfaces are covered with sandpaper of identical coarseness (P40 grade). The behavior of the mice is recorded to assess the interaction time with both textures. Mice are considered to be exploring an object when they are in its close vicinity, nose directed toward or touching the sandpaper. Resting, digging, and grooming around the object, or climbing it, is not considered as whisker-based exploration. After this first round of recognition, the mice are returned to a transport cage for 5 min. Meanwhile, one of the panels is substituted with an identical one carrying sandpaper of a finer coarseness (P100 grade) for the testing phase. The mouse is then introduced in the arena (3 min), and the total time of interaction with both textures is measured. Mice that explored the objects less than 2 s in the learning or testing phases are excluded from the analysis for a lack of exploratory activity. For this study, the fraction of excluded mice was equivalent, 3 of 10 for *AAV-flex-DTA* transduced WT, 4 of 11 for *AAV-flex-DTA Sst-Cre* mice, and 2 of 7 for non-transduced WT animals. Mice were injected with the AAV at P2 and were tested at P30 to P40.

### Fiber photometry recordings

For the injections, animals were anesthetized using isoflurane and given analgesics before surgery. A craniotomy was performed above the region of interest, and a glass pipette was stereotaxically lowered down to the desired depth. The injections were performed with a nano-inject II (Drummond Scientific). Pulses of 9.2 nl were delivered 10 s apart until the total amount was reached (120 nl). Two minutes after infusion of the entire volume, the pipette was slowly retracted. For the detection of contralateral activity, we injected* AAV2/1 CaMKII.GCaMP6f.WPRE.SV40* (Addgene, no. 100834, AAV1) into the L4 of the primary somatosensory cortex of the right hemisphere of both WT and *Sst-Cre;DTA* adult mice (7 to 12 weeks). Injection coordinates were the following (in millimeters from Bregma): AP, −0.71; ML, 3.2; and DV, 0.5. Two weeks later, animals were anesthetized using isoflurane and given analgesics to proceed to the optical ferrule implants in the contralateral (left) hemisphere. The scalp was exposed, and we applied VetbondTM (3MTM, no. 7000002814) along the cut. Then, a craniotomy was performed above the contralateral S1/S2 border, and the optical ferrule was lowered until the desired depth. Implantation coordinates were the following (in millimeters from Bregma): AP, −0.71; ML, 3.8; and DV, 0.64. Then, superglue was applied to hold the lens in position and, then, dental cement (GC FujiCEM 2) to cover the exposed skull and keep the optical ferrule in position. Animals were allowed to recover for 5 days before being used. We implanted optical ferrules with 400 μm of diameter (B280-4419-3, Doric). For fiber photometry data acquisition, the test mice were habituated to the optical fiber for 2 days by placing the fiber on the mouse head and letting it roam free for 15 min in their cage before the test. Then, we recorded the activity of the contralateral SS cortex for 5 min during the object exploration task. Movies were annotated online to simultaneously record the object interaction and the contralateral activity. We recorded the activity using a DORIC system (Basic FMC).

### Fiber photometry data analysis

We analyzed the acquired data during the entire session in each mouse. We used the Guppy software for analysis (*72*). After loading the data, we applied an artifact correction criterion consisting of the removal of the first second of the raw data. This allowed us to remove the artifacts that are usually generated right after the light sources turn on. Subtraction of the background fluorescence was calculated via a time-fitted running average of the 465-nm channel relative to the 405-nm control channel using a least squares polynomial fit of degree 1. Delta fluorescence over fluorescence (DF/F) is calculated by subtracting the fitted control channel from the signal channel and dividing by the fitted control channel using the formula (465 nm − 405 nm)/405 nm. A peak enveloping Fourier transform was applied to the DF/F signal across the entire trace to identify peaks in activity. Last, we presented the data as the deviation of the DF/F from its mean (*z*-score) using the formula (DF/F − mean of DF/F/SD of DF/F). For statistical analysis, data represents means ± SEM. We developed nested *t* test as we have multiple observations from the same mouse (from the different recording sessions).

### Object exploration in the open field

Mice were plugged into the patch cord connected to the DORIC system and then placed into the open field (60 cm by 60 cm) to habituate them to the open field for 5 min. After, we placed the mice back into their cage without disconnecting the patch cord and waited for an interval of 10 min. For the test, we placed the mice again into the open field but this time with an object placed in one of the corners and, simultaneously, recorded the contralateral activity while exploring. The recording session lasted 5 min, and FP data were acquired during the entire session. We automatically tracked the mice using the software Any-Maze 7 from Stoelting. For object interaction, we defined with the Any-Maze software a squared zone containing the object, and interaction with it was measured by the total time spent in the object zone, the number of entries in the zone, and the longest time in the zone. We also quantified the distance traveled and the time spent interacting with the object using the same automatic tracking of the test mouse (Any-Maze 7, Stoelting). We tested all the mice for at least one session and a maximum of three sessions. We waited for at least 72 hours before testing the same mice again.

### Statistical analysis

All experimental conditions include a minimum of three mice and two coronal sections per brain. Only the animals in which the IUE or the stereotaxic injections were not efficiently electroporated or with incorrect targeted areas were excluded from the analyses. Results show the sample means ± the SEM. The statistical groups were determined by genotype, treatment, area, layer, or postnatal stage. Before applying parametric statistical analysis, sample normality was tested using the D’Agostino-Pearson test. Depending on the study object and the number of variables, results were analyzed in different manners. The Student’s *t* test was used when comparing the mean values of one variable between two groups, nested *t* test for fiber photometry data in which multiple observations were obtained from the same biological sample (mouse), and a one- or two-way analysis of variance (ANOVA) for analysis of more than two groups, followed by a post hoc test, Dunnett’s, Tukey’s, or Šídák’s multiple comparisons test as specified. For electrophysiological recordings, two-group comparisons were performed using one-way ANOVA, Kolmogorov-Smirnov, Kruskal-Wallis test, or Wilcoxon matched-pairs tests depending on the normality of the distribution or two-way ANOVA for experiments involving several conditions. Statistically significant differences were established at *P < 0.05, **P < 0.01, ***P < 0.001, and ****P < 0.0001; “n.s.” stands for nonsignificant.
