# Supplementary material for: Early cortical GABAergic interneurons determine the projection patterns of L4 excitatory neurons
Source: Sci Adv. 2024 May 10;10(19):eadj9911. doi: 10.1126/sciadv.adj9911 (PMC11086621; doi:10.1126/sciadv.adj9911)
Supplement: Supplementary file 1 — Figs. S1 to S13 [file sciadv.adj9911_sm.pdf]

Science Advances
AAAS

Supplementary Materials for

Early cortical GABAergic interneurons determine the projection patterns of L4 excitatory neurons

Lorena Bragg-Gonzalo *et al.*

Corresponding author: Marta Nieto, mnlopez@cnb.csic.es

Sci. Adv. **10**, eadj9911 (2024)
DOI: 10.1126/sciadv.adj9911

This PDF file includes:

Figs. S1 to S13

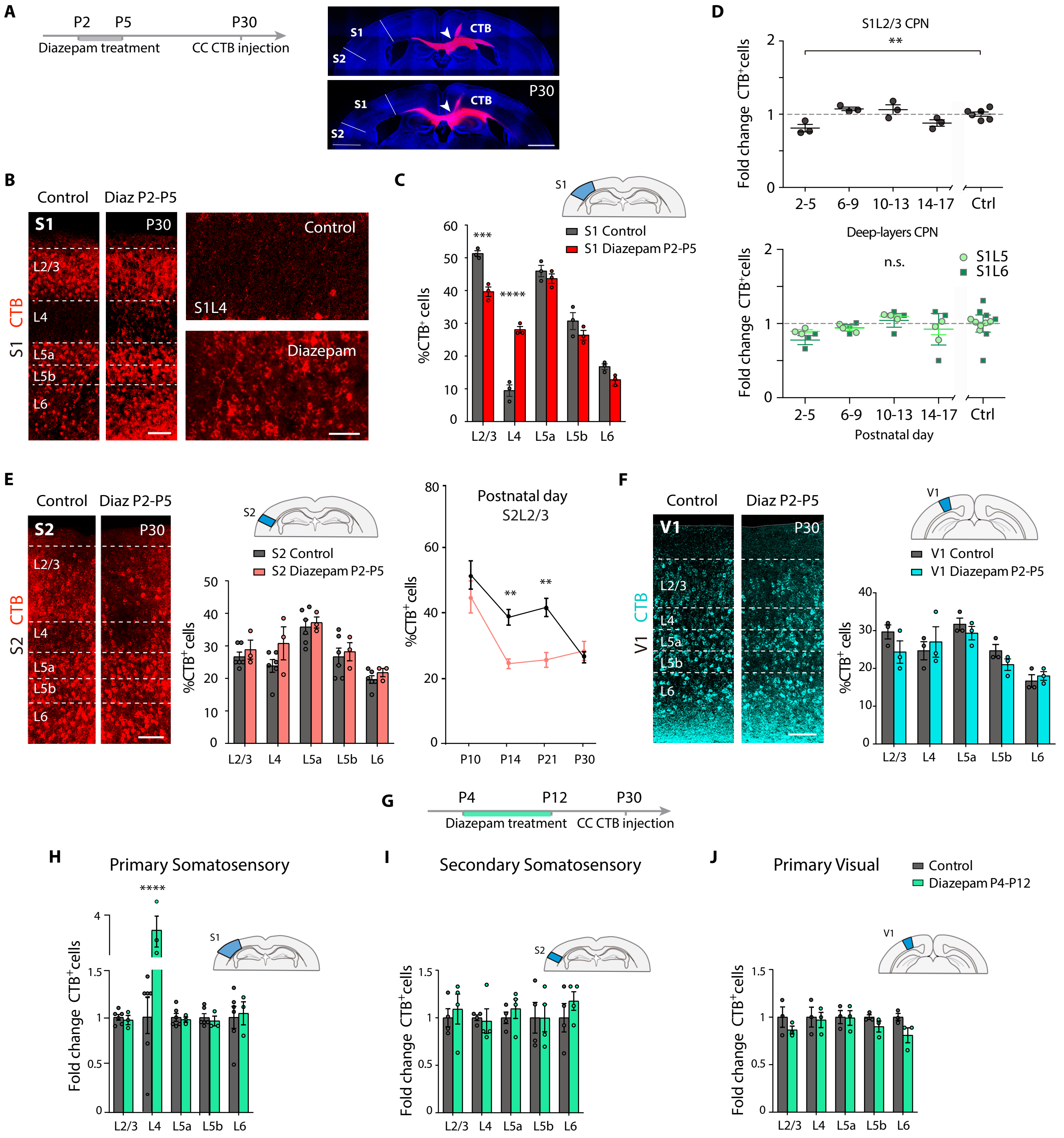

Fig. S1. Effect of various postnatal diazepam treatments. (A) Schematic of the experimental procedure in (B-F) and confocal tilescan images showing two examples of brain coronal sections 2 days post CTB injection in the CC at P30. Arrow indicates the injection site. CTB largely confines within the CC and hippocampal commissure. **(B)** Confocal images of CTB⁺ cells in S1 of P30 control and diazepam-treated (P2-P5) animals. **(C)** Quantifications of CPNs as in (B). **(D)** Number of CPNs (CTB⁺) in S1L2/3 (top) and deep layers (S1L5 and S1L6) (bottom) relative to controls upon different windows of daily diazepam treatment. **(E)** Confocal images (left) and quantifications (middle) of CTB⁺ cells in S2 of P30 control and diazepam-treated (P2-P5) animals. Analysis of S2L2/3 CTB⁺ cells at different developmental stages in control and diazepam-treated (P2-P5) animals (right). **(F)** Confocal images (left) and quantifications (right) of CTB⁺ cells in V1 of P30 control and diazepam-treated (P2-P5) animals. **(G)** Schematic of the experimental procedure to analyze the effects of a longer diazepam treatment showed in (H-J). **(H-J)** Quantifications of CTB⁺ cells in S1 (H), S2 (I) and V1 (J) of P30 control and diazepam-treated (P4-P12) animals. Data are mean ± SEM. n ≥ 3 mice per condition. **p<0.01, ***p<0.001, ****p<0.0001, n.s.=non-significant. Two-way ANOVA followed by Šídák's multiple comparison test (C, D, E, F, H, I and J). Scale bars: 500µm in (A), 100µm in (B left, E and F) and 50µm in (B right).

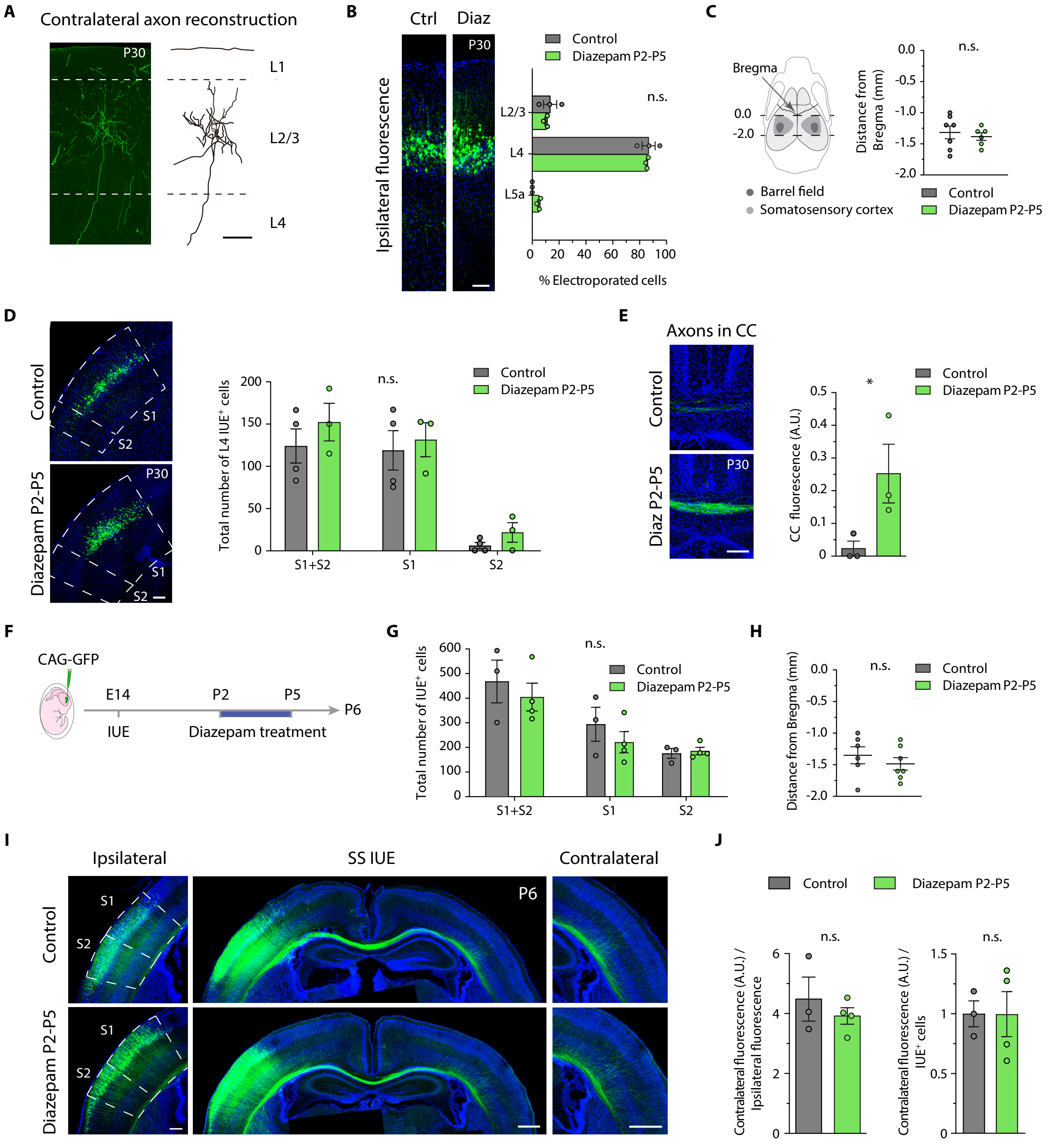

Fig. S2. Consistent and comparable IUE efficiencies for the analysis of callosal axons at P30 and at P6. (A) Representative confocal image (left) and reconstruction (right) showing the ramified terminal branching of an individual callosal axon at P30 in diazepam-treated brains. **(B)** Images of IUE$^+$ cells in a cortical column of a P30 coronal brain section of control and diazepam-treated animals (left), and quantification of the laminar distribution of fluorescent cells (right) (n > 450 cells, 3 mice per condition). **(C)** Schematic representation of the anterior-posterior levels and coordinates limiting SS areas (left). The graph represents the coordinates of the brain sections analyzed in IUE experiments for each condition (right). Both control and diazepam-treated conditions include sections of comparable anterior-posterior levels. **(D)** Tilescan images of brain sections showing equally restricted S1 location of IUE$^+$ cells in control and diazepam-treated animals. The graph shows quantifications of the total number of L4 IUE$^+$ in S1+S2 and separated individually in S1 and S2 (n > 450 cells, ≥ 3 mice per condition). **(E)** Confocal images and quantification of fluorescence at the midline confirmed that in the diazepam condition, increases in IUE$^+$ S1L4 CPNs were accompanied by a significant rise in fluorescent axons at the CC midline.

Fluorescence is normalized to the fluorescence values in the ipsilateral hemisphere. **(F)** Schematic of the experimental procedure showed in (G-J). (G-H) Analysis of electroporated neurons in P6 brains. **(G)** Indistinguishable distribution of electroporated cells in S1 and S2 areas in control and diazepam-treated brains at P6. The graph shows quantification of the total number of IUE^{+} cells in S1+S2, and in S1 and S2 separated ($n > 1000$ cells, ≥ 3 mice per condition). **(H)** Graph showing the anterior-posterior estimated position of the brain sections analyzed in (G). **(I)** Tilescan images of electroporated brain sections at P6 with magnifications of the ipsilateral and contralateral hemispheres in left and right panels, respectively. **(J)** Quantifications of contralateral fluorescence axons normalized to ipsilateral fluorescence (left) or relative to the number of ipsilateral IUE^{+} cells normalized to the control mean (right) ($n \geq 3$ mice). A.U.= arbitrary units. Data are mean ± SEM. Dots represent mean values of individual mice (B, D, E, G and J) or brain sections (C and H). *$p<0.05$, n.s.=non-significant. Two-way ANOVA followed by Šídák's multiple comparison test in (B, D, G) and unpaired t-test in (C, E, H and J). Scale bars: 100µm in (D, E and I), 50 µm in (B) and 10µm in (A).

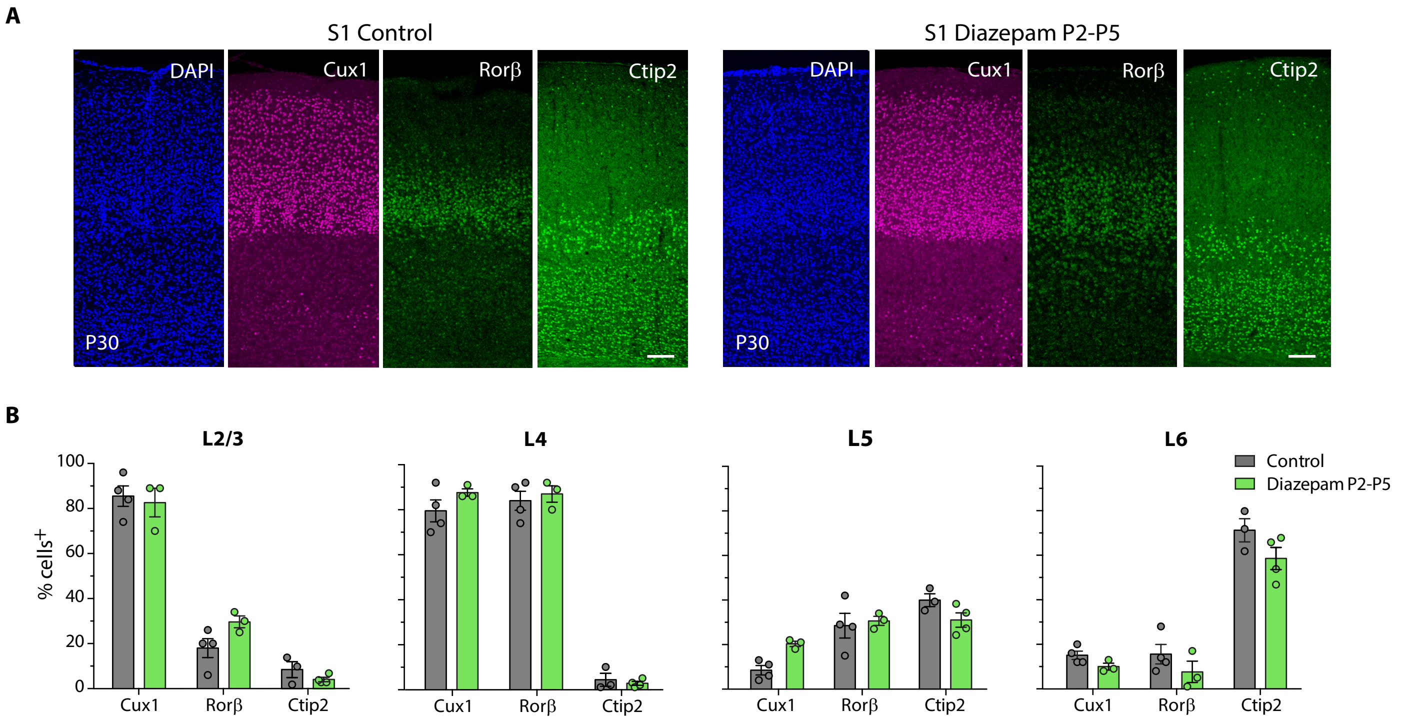

Fig. S3. No changes in the expression of laminar molecular markers upon early diazepam treatment. (A) Confocal images of DAPI, Cux1, Rorβ and Ctip2 immunostainings in P30 histological sections of control (left) and diazepam-treated (right) brains. **(B)** Quantification of cells expressing the indicated molecular marker expressed as percentage of DAPI$^+$ nuclei (n ≥ 3 mice per condition). Data showed no statistical differences for any marker. Data are mean ± SEM. Two-way ANOVA followed by Šídák's multiple comparison test in (B). Scale bars: 100µm in (A).

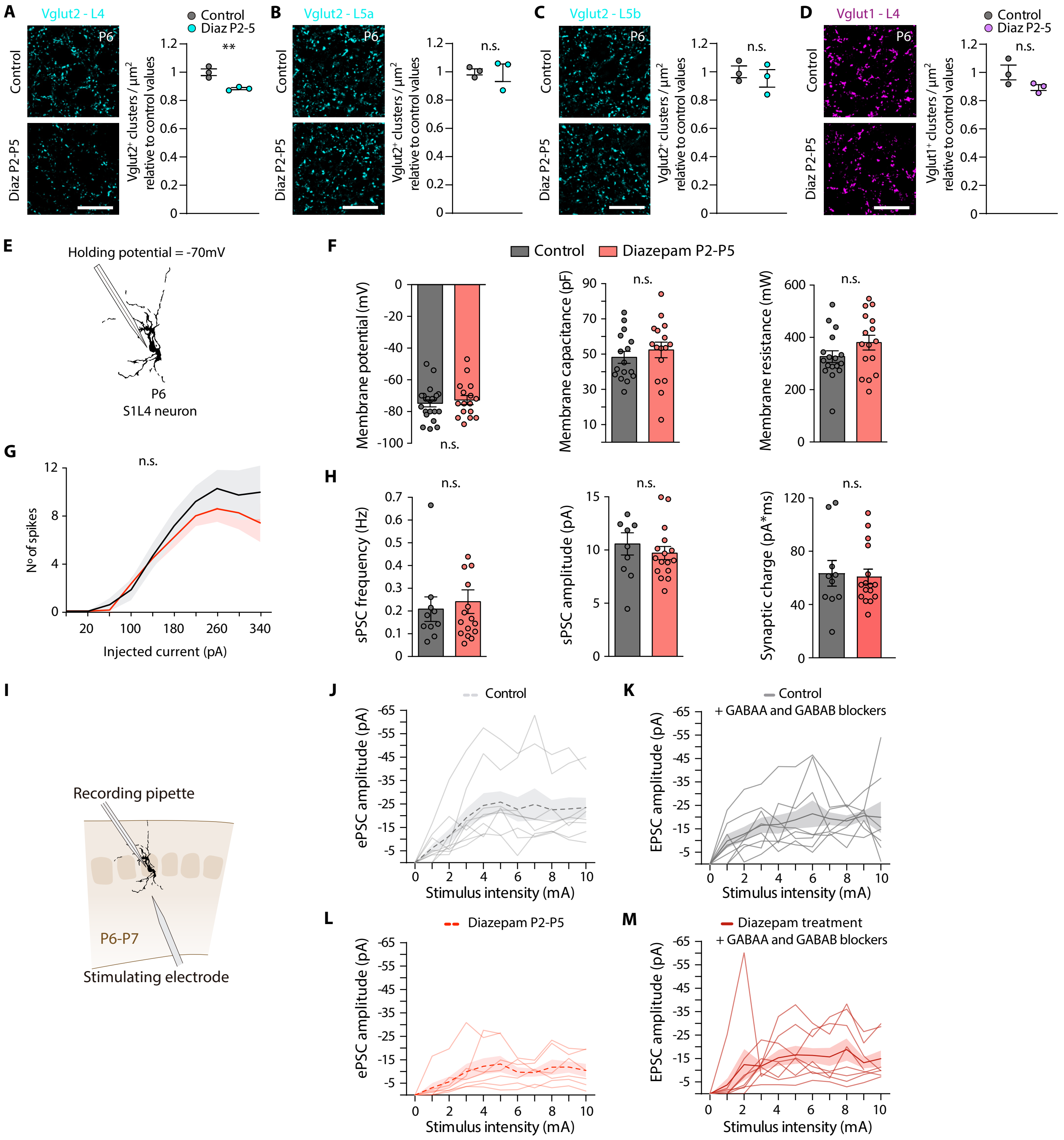

Fig. S4. Analysis of thalamic inputs and functional connectivity of P6-P7 S1L4 PNs after P2-P5 diazepam treatment. (A) Images (left) and quantifications (right) of Vglut2^{+} clusters in S1L4 at P6. Diazepam-treated animals have reduced bouton density compared to controls. **(B-C)** Images (left) and quantifications (right) showing no differences in the density of Vglut2^{+} clusters in S1L5a (A) or S1L5b (B) in P6 diazepam-treated brains compared to controls. **(D)** Images (left) and analysis (right) of Vglut1^{+} inputs in S1L4 also shows no changes between conditions. Dots show average value per mouse (n=3 mice per condition). **(E)** Scheme depicting whole-cell patch-clamp recording. **(F)** Analysis of passive membrane properties of P6-P7 S1L4 PNs after animals were treated with diazepam between P2-P5 (n ≥ 15 cells; n=3 mice per condition). **(G)** Intrinsic firing responses of P6-P7 S1L4PNs (n ≥ 12 cells per condition). **(H)** The frequency, amplitude and synaptic charge of spontaneous synaptic events were indistinguishable in diazepam-treated and control mice. Each dot represents an individual recorded S1L4 neuron (n ≥ 9 cells; n=3 mice per condition). **(I-M)** Deep-layer stimulation paradigm and individual responses of the recorded neurons (from Fig. 2, G and H). Evoked postsynaptic currents=ePSC, evoked excitatory postsynaptic currents=EPSC. Data are mean ± SEM. Solid lines represent mean and shaded area ± SEM. **p<0.01, n.s.=non-significant. Two-way ANOVA followed by Šídák's multiple

comparison test in (G) and unpaired t-test in (A, B, C, D, F and H). Scale bars: 15μm in (A, B, C and D).

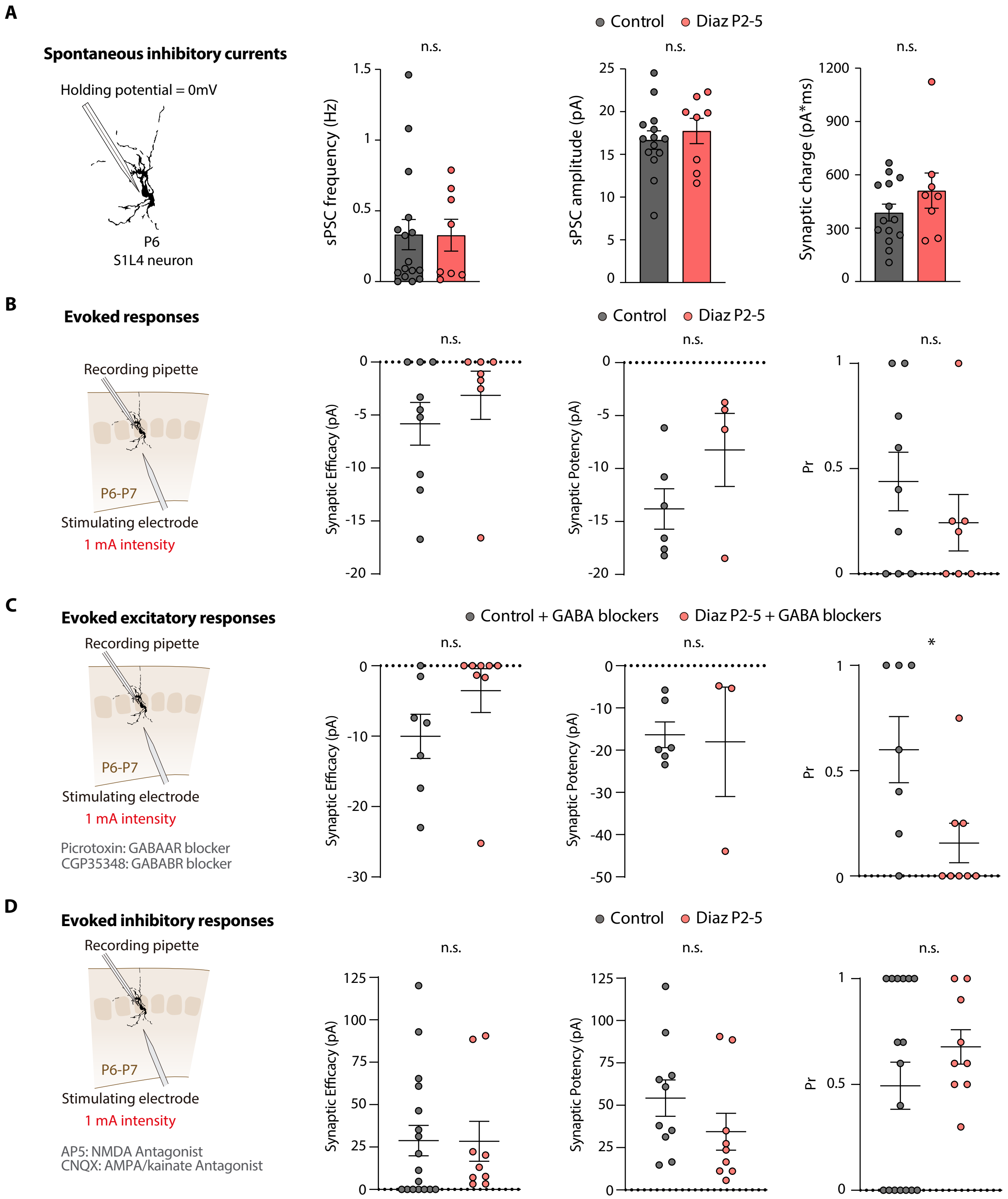

Fig. S5. Functional connectivity of P6-P7 S1L4 PNs at minimal stimulation intensities after P2-P5 diazepam treatment. (A) Spontaneous synaptic events for inhibitory currents showing no changes between conditions (n ≥ 8 cells; n=3 mice per condition). **(B)** 1mA deep-layer stimulation paradigm and individual responses of S1L4 recorded neurons. Synaptic efficacy (average of all responses including failures), synaptic potency (amplitude of responses) and synaptic failure, Pr (ratio of responses from the total of stimuli applied) showed no differences in control and diazepam-treated mice. 1mA=minimal intensity applied with the stimulation electrode (n ≥ 7 cells; n=3 mice per condition). **(C)** Quantifications of S1L4 responses as in (B) in the presence of GABAR blockers (n ≥ 7 cells; n=3 mice per condition). **(D)** Quantifications of S1L4 responses as in (B) in the presence of NMDA and AMPA/kainite blockers to record inhibitory responses (n ≥ 9 cells; n=3 mice per condition). Spontaneous postsynaptic currents=sPSC. Each dot represents an individual recorded S1L4 neuron. Data are mean ± SEM. *p<0.05, n.s.=non-significant. Unpaired t-test in (A, B, C and D).

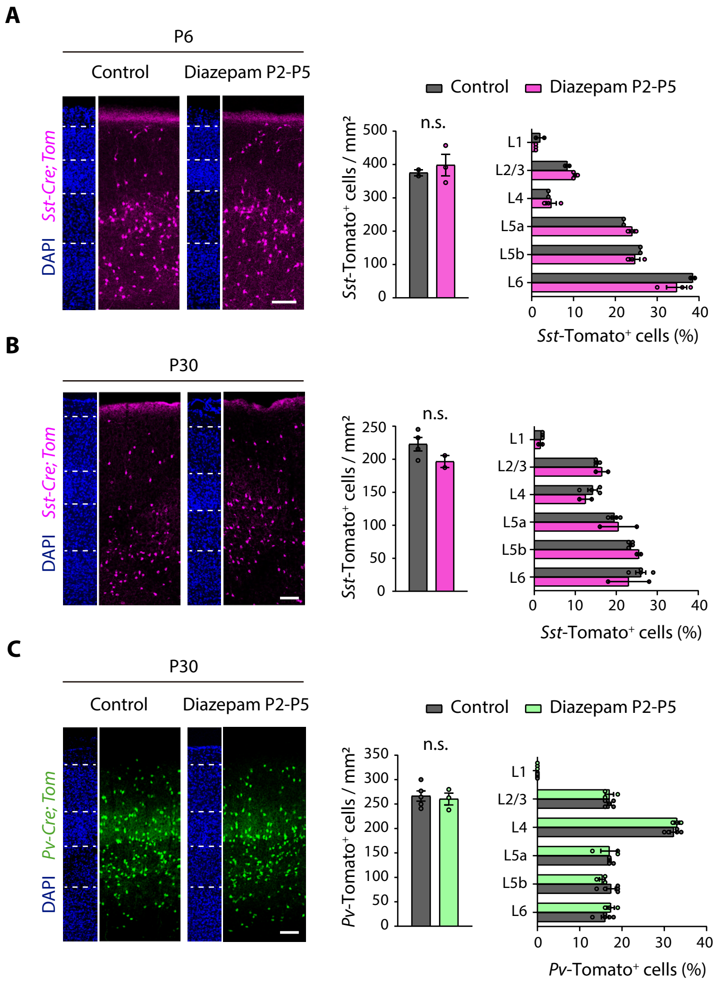

Fig. S6. P2-P5 diazepam treatment does not alter MGE-derived interneuron number. (A) Confocal images in S1 of Tom+ cells in P6 *Sst-Cre;Tom* mice (left). Quantifications of total *Sst*-Tom+ cell density in the cortical column (middle) and in each cortical layer (right) comparing control and diazepam-treated mice. **(B)** Same as in (A) in P30 *Sst-Cre;Tom* mice. No differences in SST-IN density were observed at both stages in diazepam-treated animals. **(C)** Confocal images in S1 of Tom+ cells in P30 *Pv-Cre;Tom* mice (left). Quantifications of total *Pv*-Tom+ cell density in the cortical column (middle) and in each cortical layer (right) comparing control and diazepam-treated mice. Treatment also did not change PV-IN number. Data are mean ± SEM. n ≥ 2 mice per condition. n.s.=non-significant. Two-way ANOVA followed by Šídák's multiple comparison test in (A right, B right and C right) and unpaired t-test in (A middle, B middle and C middle). Scale bar: 100µm in (A, B and C).

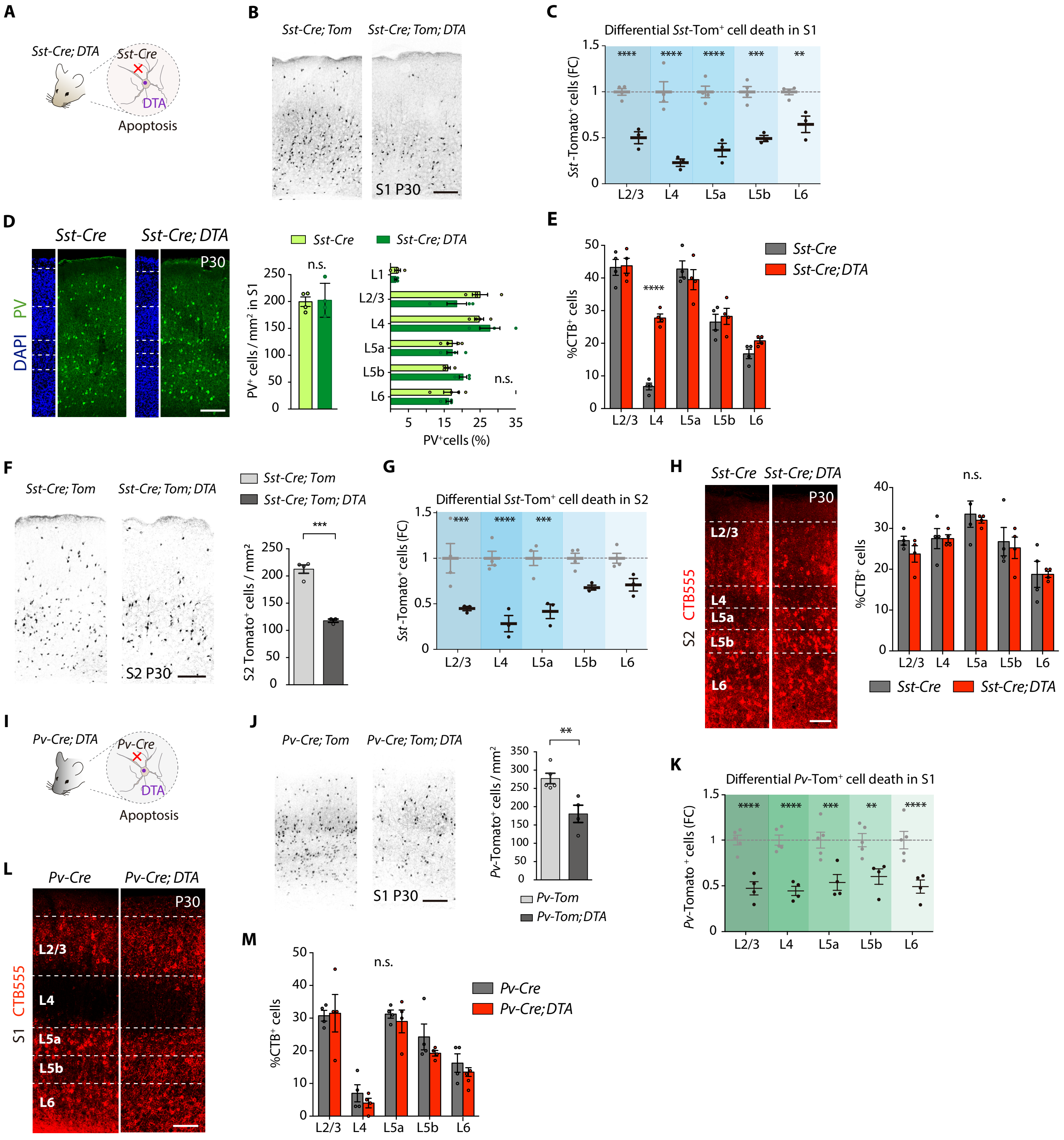

Fig. S7. Selective ablation of SST-INs rewires S1L4 (but not S2L4) into CPNs. The efficiency of SST-IN deletion was validated by crossing *Sst-Cre;DTA* mice with a floxed *tdTomato* (*Tom*) reporter mice (*Sst-Cre;DTA;Tom*). Quantifications in these mice showed that DTA expression eliminates half of the Tom^{+} cells in S1 while parvalbumin immunostaining showed that PV-INs remained unaffected. **(A)** Scheme of SST-INs genetic ablation. **(B)** Images and **(C)** quantification at P30 of the loss of SST-INs in S1of *Sst-Cre;DTA;Tom* mice compared to *Sst-Cre;Tom* animals per cortical layer (n ≥ 3 mice). Data are normalized to *Sst-Cre;Tom* control values. **(D)** Parvalbumin immunostaining (left) and PV-INs quantification (middle and right) in *Sst-Cre* and *Sst-Cre;DTA* mice (n ≥ 3 mice). **(E)** Quantification of CPNs (%CTB^{+}/ DAPI^{+}) in *Sst-Cre* or *Sst-Cre;DTA* mice showing increased numbers in S1L4 in the latter (n = 4 mice per condition). **(F)** Images and quantification at P30 of the loss of SST-INs in S2 of *Sst-Cre;DTA;Tom* mice compared to *Sst-Cre;Tom* animals (n ≥ 3 mice). **(G)** SST-IN numbers per layer. Data is normalized to control values of *Sst-Cre;Tom* mice (n ≥ 3 mice). **(H)** Confocal images and quantifications of CPNs in S2. No differences between conditions are observed (n = 4 mice per condition). (I-M) Genetic ablation of PV-INs using *Pvalb* promoter does

not alter CPNs. **(I)** Scheme of PV-INs genetic ablation. **(J)** Images and quantification of the loss of PV-INs at P30 in S1 of *Pv-Cre;DTA;Tom* mice compared to *Pv-Cre;Tom* animals (n ≥ 4 mice). **(K)** PV-IN numbers per layer. Data is normalized to control values of *Pv-Cre;Tom* mice (n ≥ 4 mice). **(L)** Confocal images and **(M)** quantifications of CPNs (% CTB^+/ $DAPI^+$) in S1 of *Pv-Cre* and *Pv-Cre;DTA* mice (n = 4 mice per condition). Data are mean ± SEM. **p<0.01, ***p<0.001, ****p<0.0001, n.s.=non-significant. Two-way ANOVA followed by Šídák's multiple comparison test in (C, D right, E, G, H, K and M) and unpaired t-test in (D middle, F and J). Scale bar: 100µm in (B, D, F, H, J and L).

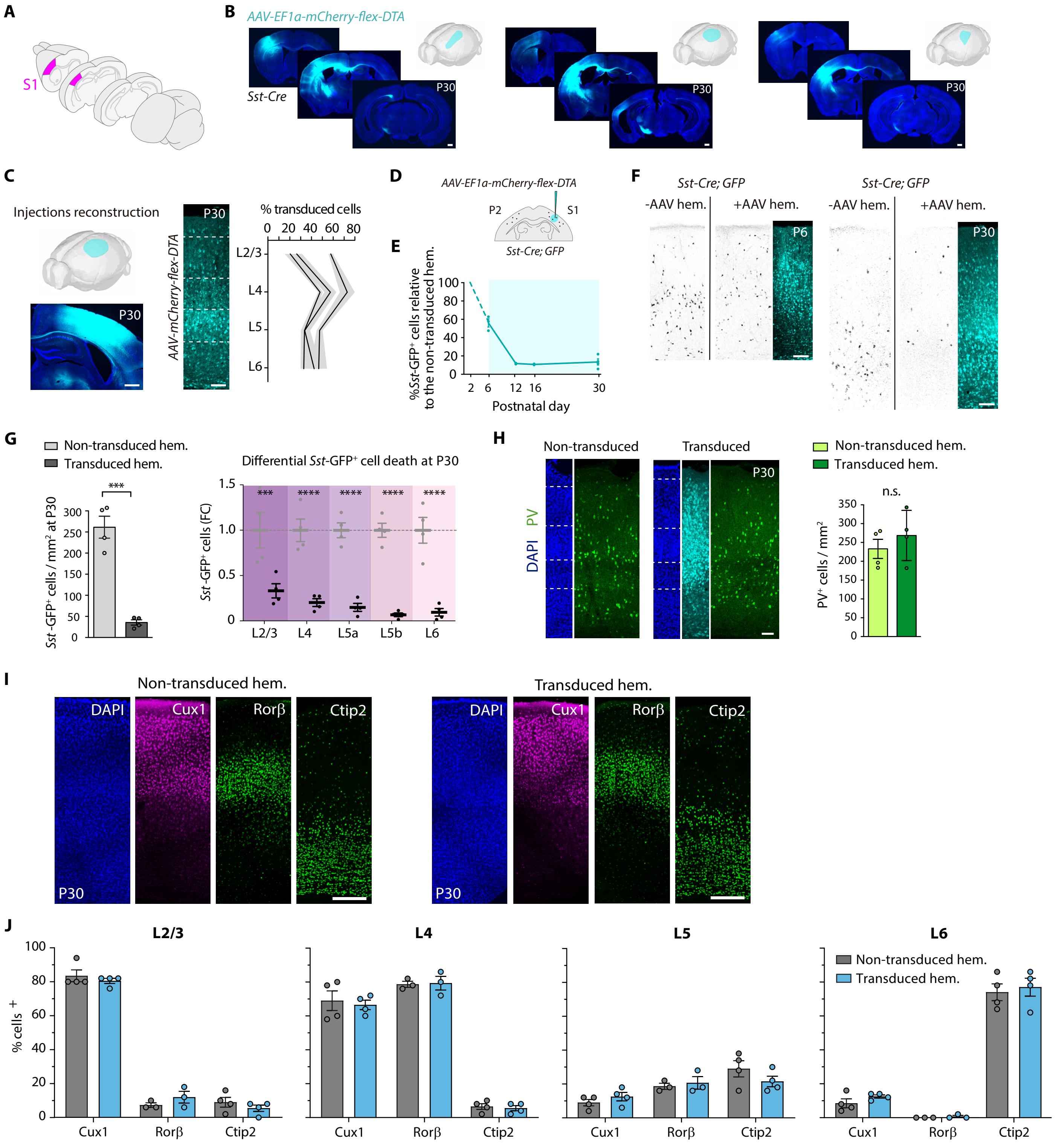

Fig. S8. Efficient elimination of SST-INs using *AAV8-EF1α-mCherry-flex-DTA* injected in *Sst-Cre* mice. S1 injections of the adeno-associated viral vector (AAV) containing a flex-DTA and mCherry as a transduction marker (*AAV-EF1α-mCherry-flex-DTA*) in *Sst-Cre* mice. Transductions were performed by injecting the S1 cortex at P2 to analyze the contribution of SST-IN cortical population. **(A)** Schematic representation of the injection site. **(B)** 3D reconstructions of the virus transduction spreading in P30 brains informed by the mCherry reporter expressed in the AAV (cyan-coloured). mCherry labels somas in the transduced area and projections, such as callosal and pyramidal tract axons. **(C)** Quantifications of the percentage of mCherry+ transduced cells in each layer (n = 3 mice). Images in (B) and analysis in (C) confirmed correct S1 targeting and comparable transductions, with high efficiencies in all injected brains. **(D)** Schematic representation of the experiment to quantify the efficiency of SST-IN elimination by injecting the *AAV-EF1α-mCherry-flex-DTA* in a floxed *GFP* reporter mice (*Sst-Cre;GFP*). **(E)** Quantification of SST-IN loss at sequential postnatal stages after injections at P2. We found a ~50% reduction in *Sst*-GFP+ cells at P6 (four days after the injection), and almost complete elimination at P12 and thereafter (n ≥ 2 mice at

each developmental stage). **(F)** Images of P6 and P30 *Sst-Cre;GFP* brain coronal sections showing *Sst*-GFP$^+$ cells after *AAV-EF1*α*-mCherry-flex-DTA* transduction at P2. **(G)** Density of *Sst*-GFP$^+$ neurons in S1 area of P30 brains injected at P2. Comparison between non-transduced and transduced hemispheres in the entire S1 cortical column (left panel) and in each S1 cortical layer (right panel, relative to non-transduced hemisphere) (n = 4 mice). **(H)** Unchanged numbers of PV-INs confirmed the specificity of the deletion using the *AAV-EF1*α*-mCherry-flex-DTA* injected in *Sst-Cre* mice. Confocal images (left) and quantification of immunopositive PV cell density (right) in S1 in non-transduced and transduced hemispheres (n = 4 mice). **(I)** Confocal images of DAPI, Cux1, Rorβ and Ctip2 immunostainings in P30 histological sections of non-transduced (left) and transduced (right) *Sst-Cre* brains. **(J)** Quantification of cells expressing the indicated molecular marker expressed as percentage of DAPI$^+$ nuclei (n ≥ 3 mice). Data showed no statistical differences for any marker. Data are mean ± SEM. Solid lines represent mean and shaded area ± SEM. ***p<0.001, ****p<0.0001, n.s.=non-significant. Two-way ANOVA followed by Šídák's multiple comparison test in (G right and J) and unpaired t-test in (G left and H). Scale bar: 500µm in (B and C left) and 100 µm in (C right, F, H and I).

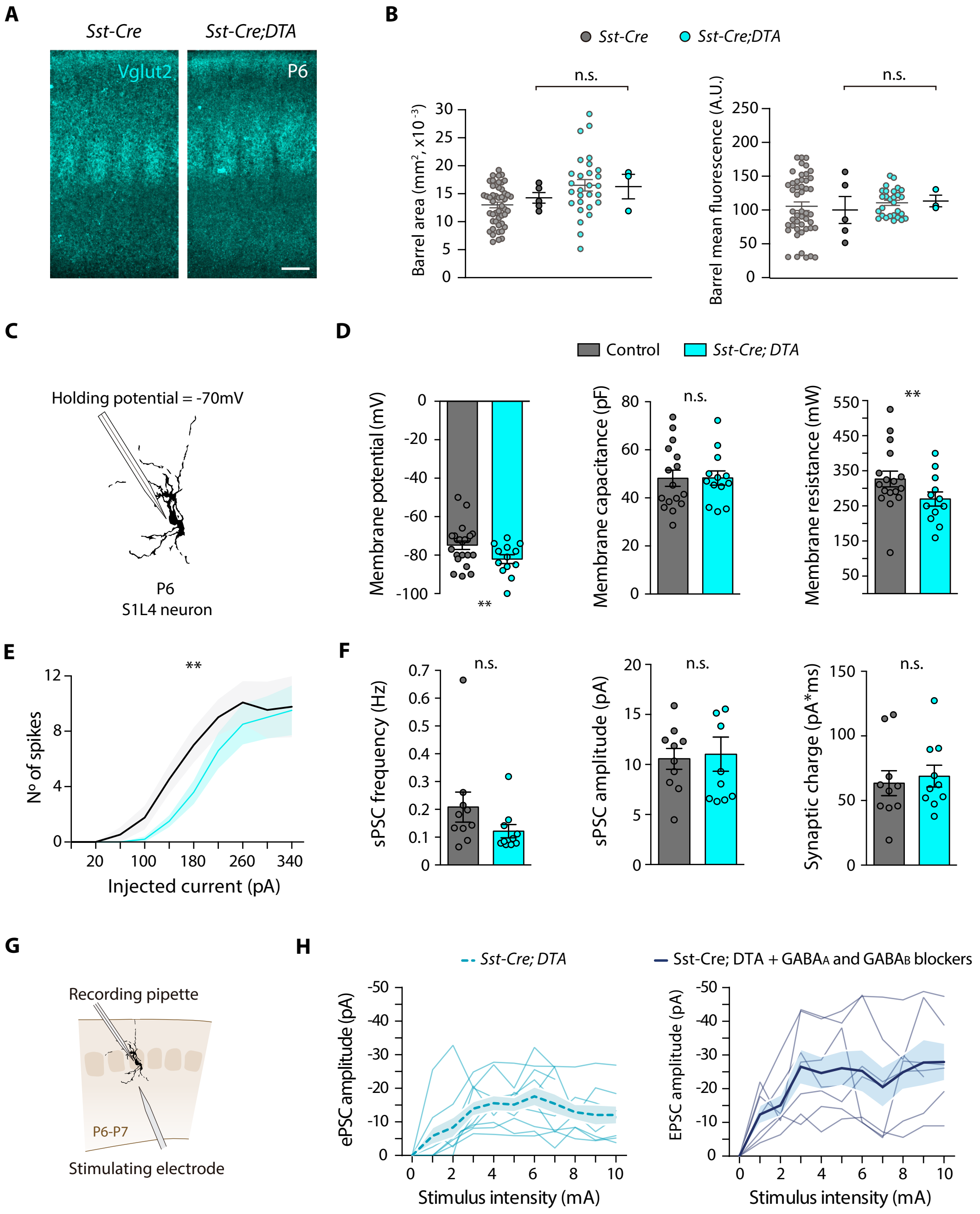

Fig. S9. Electrophysiological recordings from P6-P7 S1L4 PNs in control and *Sst-Cre;DTA* mice. (A-B) Confocal images of Vglut2 immunostaining (A) and analysis (B) of individual barrel area and mean fluorescence in the BF of *Sst-Cre* and *Sst-Cre;DTA* mice at P6. Small dots represent individual barrel values (left) whereas big dots (right) show average value per mouse (n ≥ 28 barrels, ≥ 3 mice per condition). **(C)** Scheme depicting whole-cell patch-clamp recording in a S1L4 neuron for analysis of intrinsic responses. **(D)** Intrinsic properties of P6-P7 S1L4 PNs in control versus SST- IN ablated cortices. Whole-cell recordings of S1L4 PNs of *Sst-Cre;DTA* mice showed significantly hyperpolarized membrane potential and reduced membrane resistance when compared to controls (n ≥ 16 cells in control and n ≥ 12 in mutants; n=3 mice per condition). **(E)** Firing responses to cell-injected currents. S1L4 PNs of *Sst-Cre;DTA* mice showed reduced firing compared to controls. These features indicate reduced intrinsic excitability and are suggestive of an immature electrophysiological state of S1L4 PNs upon SST-INs ablation (n = 13 cells in control and n = 12 in mutants; n=3 mice per condition). **(F)** Frequency, amplitude and synaptic charge of spontaneous postsynaptic currents showed indistinguishable values between conditions (n = 10 cells in control and n ≥ 9 in mutants; n=2 mice per condition). **(G)** Scheme of deep-layer stimulation and whole-cell patch-clamp recording in a S1L4 neuron. **(H)** Individual responses of the recorded S1L4 neurons (from Fig. 4, B and D). Evoked postsynaptic currents=ePSC, evoked excitatory postsynaptic currents=EPSC. A.U.= arbitrary units. Data are mean ± SEM. Solid lines represent mean and shaded area ± SEM. **p<0.01, n.s.=non-significant. Two-way ANOVA followed by Šídák's multiple comparison test in (E) and unpaired t-test in (B, D and F). Scale bars: 100µm in (A).

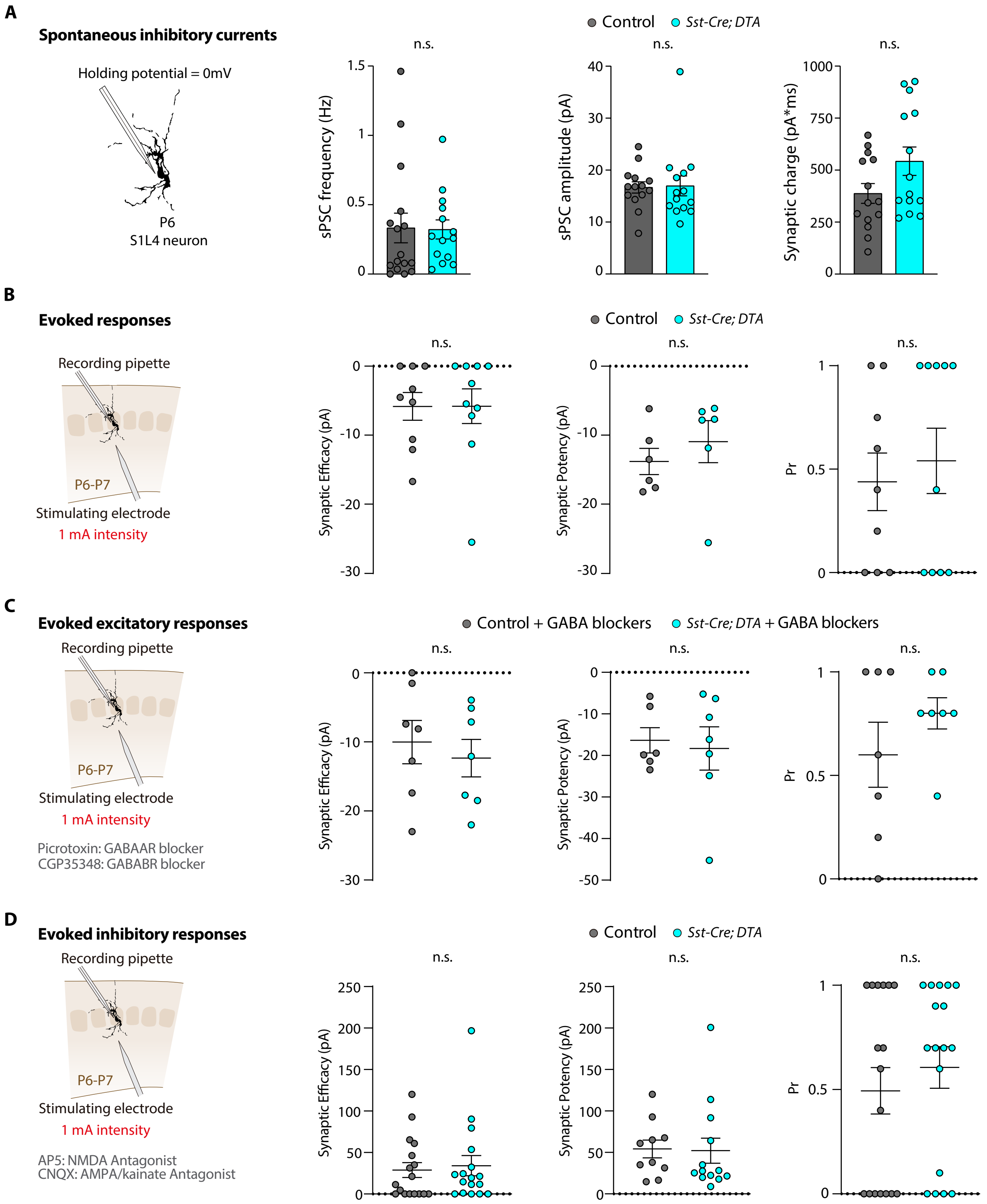

Fig. S10. Functional connectivity of P6-P7 S1L4 PNs at minimal stimulation intensities in control and *Sst-Cre;DTA* mice. (A) Spontaneous synaptic events for inhibitory currents. No differences were recorded between conditions (n ≥ 14 cells; n=3 mice per condition). **(B)** 1mA deep-layer stimulation paradigm and individual responses of S1L4 recorded neurons. Synaptic efficacy, synaptic potency and synaptic failure remained unchanged in control and *Sst-Cre;DTA* mice. 1mA=minimal intensity applied with the stimulation electrode (n ≥ 9 cells; n=3 mice per condition). **(C)** Quantifications of S1L4 responses as in (B) in the presence of GABAR blockers (n = 7 cells; n=3 mice per condition). **(D)** Quantifications of S1L4 responses as in (B) in the presence of NMDA and AMPA/kainite blockers to measure inhibitory responses (n = 17 cells; n=3 mice per condition). Spontaneous postsynaptic currents=sPSC. Each dot represents an individual recorded S1L4 neuron. Data are mean ± SEM. n.s.=non-significant. Unpaired t-test in (A, B, C and D).

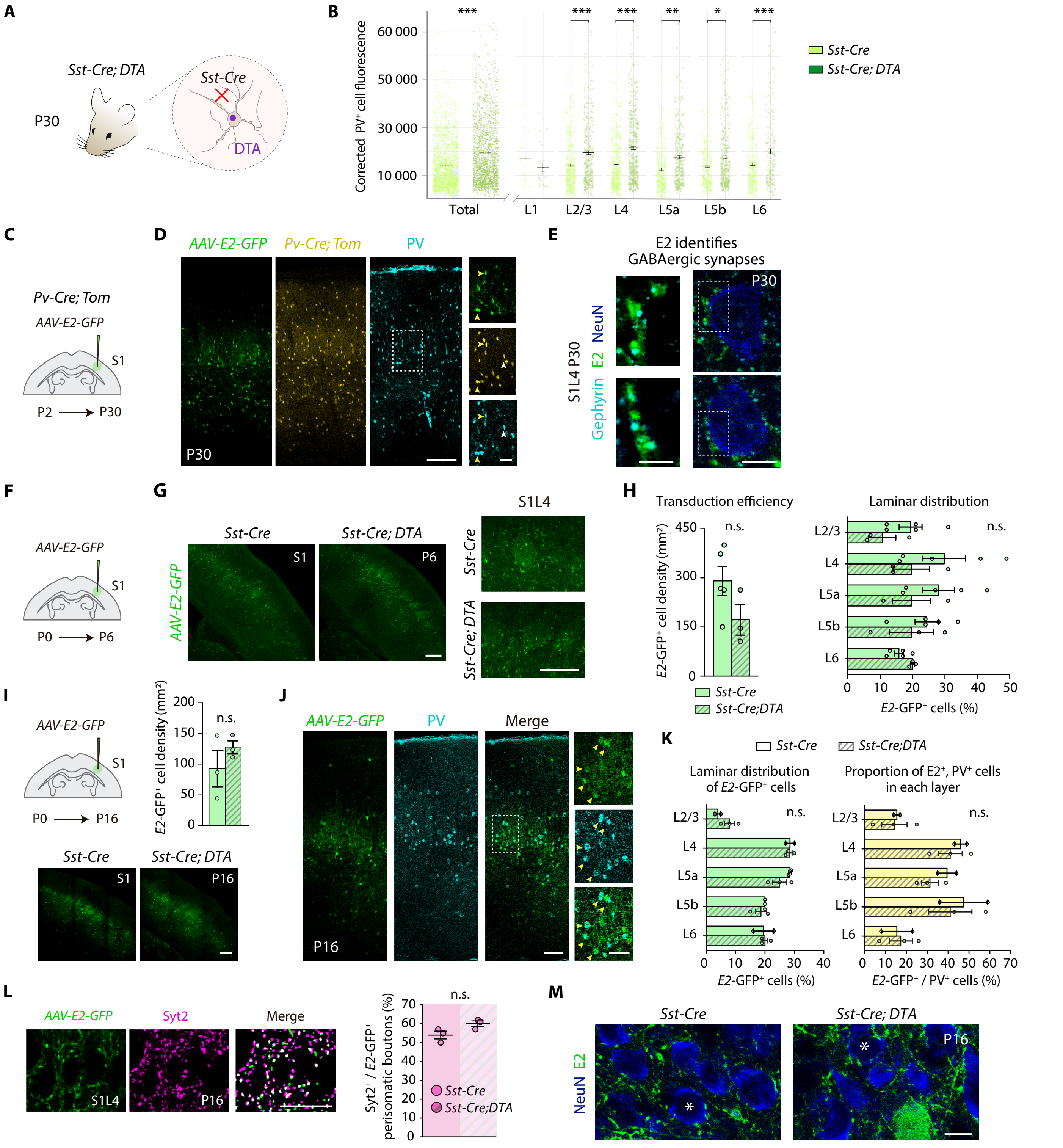

Fig. S11. Characterization of *E2* enhancer and viral injection efficiencies for the analysis of PV-mediated inhibition. (A) Scheme of SST-IN genetic ablation. **(B)** Analysis at P30 of fluorescence intensity of immunopositive PV cells in S1 (graph, left) and in each S1 layer (graph, right), in *Sst-Cre* control and *Sst-Cre;DTA* mutant mice. The corrected cell fluorescence (CTCF) of each cell is calculated by subtracting background signal (value of integrated density of the cell minus the product of its area and mean background fluorescence in the sample) (n = 2013 PV+ cells in control and n = 1886 PV+ cells in mutants; n=3 mice per condition). Each dot represents an individual PV+ cell. **(C)** Scheme of the experimental procedure in (D-E). *AAV-E2-GFP* was injected at P2 and the brains were analyzed at P30. **(D)** Confocal images of S1 cortex of *Pv-Cre;Tom* mice transduced with the *AAV-E2-GFP* at P2 and analyzed at P30. Representative images of *E2*-GFP+ transduced cells, *Pv*-Tom+ cells, and immunopositive PV neurons. Quantifications are shown in (Fig. 5B). **(E)** Detail of perisomatic GABAergic synapses in P30 S1L4 neurons composed by the apposition of presynaptic *E2*-GFP+ boutons and the postsynaptic marker gephyrin. **(F)** Scheme of the experimental procedure in (G-H). *AAV-E2-GFP* was injected at P0 and the

brains were analyzed at P6. **(G)** Confocal images of *E2*-GFP$^+$ signal in S1 and higher magnifications of S1L4 transduced neurons at P6. **(H)** Quantifications of the density (left) and layer distribution (right) of *E2*-GFP$^+$ cells (n = 5 mice in *Sst-Cre* and n = 3 mice in *Sst-Cre;DTA*). Each dot represents the value of individual mice. **(I)** Scheme of the experimental procedure in (J-M). *AAV-E2-GFP* was injected at P0 and the brains were analyzed at P16 (left). Quantification of the transduction efficiency at P16 (right) and confocal images of the resulting transduction (lower panels) (n = 3 mice in *Sst-Cre* and n = 3 mice in *Sst-Cre;DTA*). Each dot represents the value of individual mice. **(J)** Confocal images of S1 transduced with the *AAV-E2-GFP* at P0 and analyzed at P16. Representative images of *E2*-GFP$^+$ transduced cells and immunopositive PV neurons. **(K)** Graphs showing the laminar distribution of *E2*-GFP$^+$ cells (left) and the proportion of double positive cells (*E2*-GFP$^+$ and PV$^+$) in each layer (right) (n = 2 mice in *Sst-Cre* and n = 3 mice in *Sst-Cre;DTA*). Each dot represents the value of individual mice. **(L)** Confocal images (left) of P16 brains showing transduced *E2*-GFP$^+$ and immunolabelled Syt2$^+$ boutons in S1L4. Graph (right) shows the percentage of colocalization between *E2*-GFP$^+$ and Syt2$^+$ boutons onto S1L4 NeuN$^+$ somas (n = 3 mice in *Sst-Cre* and n = 3 mice in *Sst-Cre;DTA*). Each dot represents the value of individual mice. **(M)** Images of *E2*-GFP$^+$ perisomatic boutons in P16 *Sst-Cre* and *Sst-Cre;D-TA* brains. Quantification shown in (Fig. 5G). Synaptotagmin-2=Syt2. Data are mean ± SEM. *p<0.05, **p<0.01, ***p<0.001, n.s.=non-significant. Two-way ANOVA followed by Šídák's multiple comparison test in (H right and K) and unpaired t-test in (B, H left, I and L). Scale bars: 200µm in (D left, G left, I), 100µm in (J left), 50µm in (D right, G right, J right), 10µm in (L and M) and 2.5µm in (E).

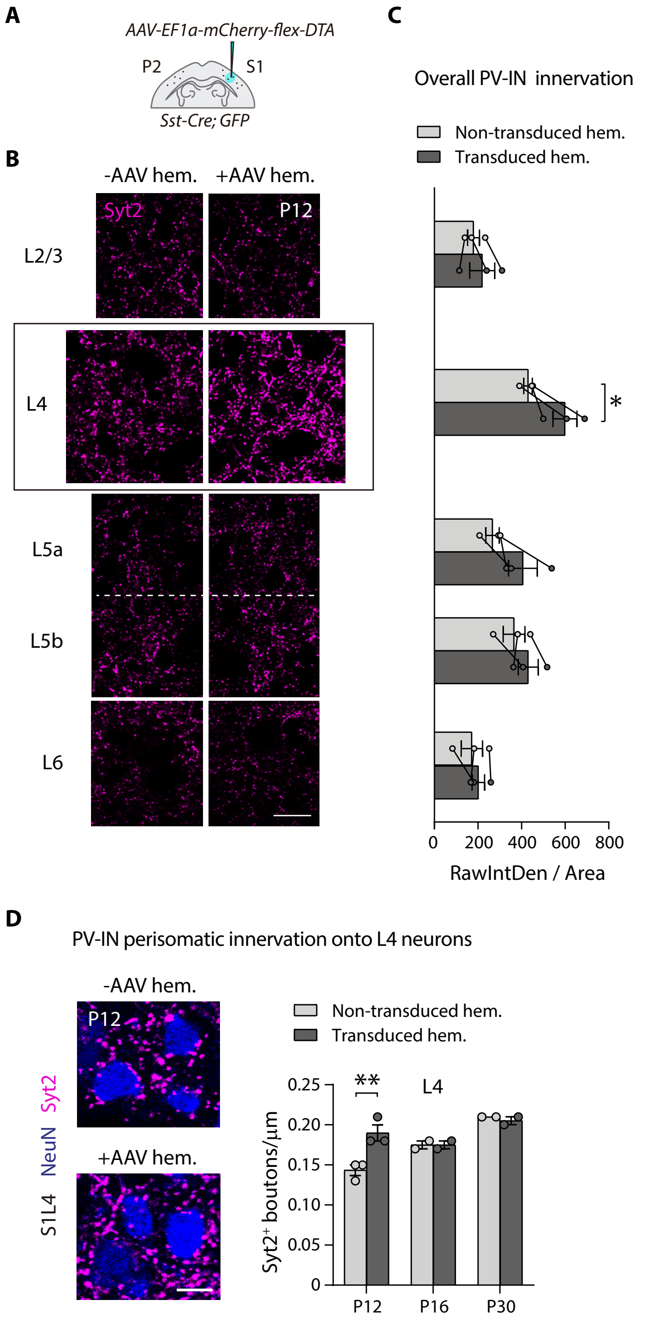

Fig. S12. Characterization of PV-IN innervation upon AAV-mediated ablation of SST-INs. (A) Schematic representation of SST-IN depletion using the *AAV-EF1α-mCherry-flex-DTA* injected at P2 in *Sst-Cre* mice. **(B)** Confocal images showing Syt2 immunostaining throughout S1 cortical layers. **(C)** Quantification of the overall Syt2 boutons measured by normalizing the raw integrated density (RawIntDen) by the ROI area (n = 3 mice). **(D)** Detail of P12 S1L4 Syt2 staining (left) and quantifications (right) of Syt2 perisomatic bouton density at P12, P16 and P30 (n = 3 mice for P12 data, n = 2 mice for P16 data, n = 2 mice for P16 data). Data are mean ± SEM. Each dot represents an individual mouse. *p<0.05, **p<0.01, n.s.=non-significant. Unpaired t-test in (C and D). Scale bars: 25μm in (B) and 10μm in (D).

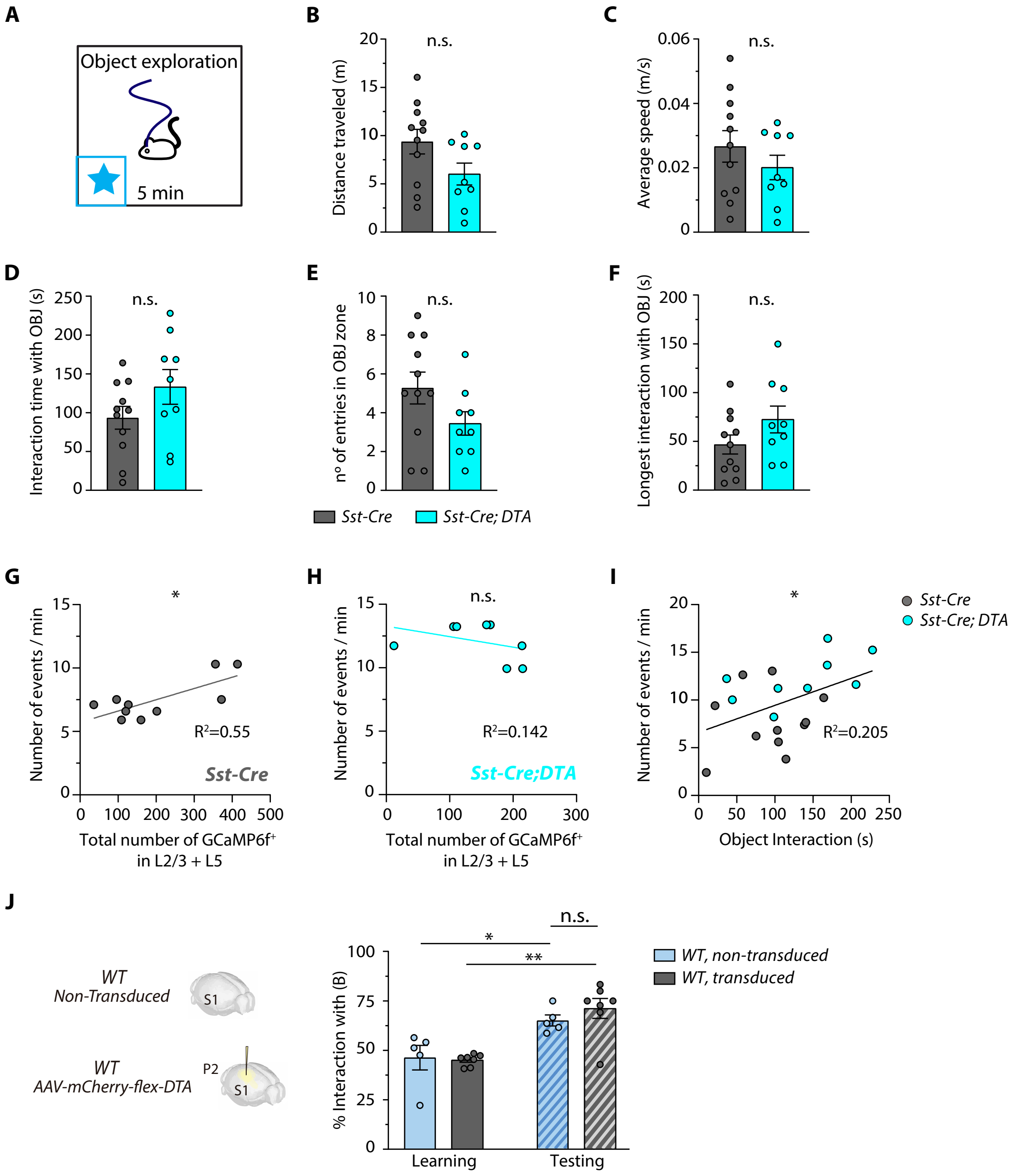

Fig. S13. *Sst-Cre;DTA* mice show no behavioral differences during exploration. (A) Schematic of the behavioral paradigm. Freely moving animals performed a 5-min object exploration task. **(B-C)** Locomotion parameters (i.e distance traveled (B) and average speed (C)) showed no changes between control *Sst-Cre* and mutant *Sst-Cre;D-TA* mice (n = 5 mice in *Sst-Cre*, n = 4 mice in *Sst-Cre;DTA*, ≥ 2 sessions per mouse). **(D-F)** Exploratory behaviors (i.e. interaction time with the object (D), number of entries in the object zone (E) and longest interaction time with the object (F)) were also unaffected between conditions (n = 5 mice in *Sst-Cre*, n = 4 mice in *Sst-Cre;DTA*, ≥ 2 sessions per mouse). **(G-H)** Pearson correlation analysis of GCaMP6f⁺ L2/3 and L5 cell number per average mean frequency in its contralateral hemisphere. We found a significant correlation in *Sst-Cre* (G), not observed in mutant *Sst-Cre;DTA* mice (H) (n = 5 mice in *Sst-Cre*, n = 4 mice in *Sst-Cre;DTA*, ≥ 2 sessions per mouse). **(I)** Pearson correlation analysis demonstrating a whisker sensory dependency of calcium contralateral transients (n = 5 mice in *Sst-Cre*, n = 4 mice in *Sst-Cre;DTA*, ≥ 2 sessions per mouse). **(J)** Scheme depicting the experimental approach (left) and quantification (right) of time percentage of interaction with the B texture in the learning and testing phases comparing AAV-transduced and non-transduced WT animals (n = 5 mice in non-transduced, n = 7 mice in transduced). Data are mean ± SEM. *p<0.05, **p<0.01, n.s.=non-significant. Nested t-test in (B, C, D, E and F), Pearson r correlation in (G, H and I) and one-way ANOVA followed by Šídák's multiple comparison test in (J).
